# The long-range interaction map of ribosomal DNA arrays

**DOI:** 10.1371/journal.pgen.1007258

**Published:** 2018-03-23

**Authors:** Shoukai Yu, Bernardo Lemos

**Affiliations:** Program in Molecular and Integrative Physiological Sciences & Department of Environmental Health, Harvard T. H. Chan School of Public Health, Boston, MA, United States of America; Geisel School of Medicine at Dartmouth, UNITED STATES

## Abstract

The repeated rDNA array gives rise to the nucleolus, an organelle that is central to cellular processes as varied as stress response, cell cycle regulation, RNA modification, cell metabolism, and genome stability. The rDNA array is also responsible for the production of more than 70% of all cellular RNAs (the ribosomal RNAs). The rRNAs are produced from two sets of loci: the 5S rDNA array resides exclusively on human chromosome 1 while the 45S rDNA arrays reside on the short arm of five human acrocentric chromosomes. These critical genome elements have remained unassembled and have been excluded from all Hi-C analyses to date. Here we built the first high resolution map of 5S and 45S rDNA array contacts with the rest of the genome combining over 15 billion Hi-C reads from several experiments. The data enabled sufficiently high coverage to map rDNA-genome interactions with 1MB resolution and identify rDNA-gene contacts. The map showed that the 5S and 45S arrays display preferential contact at common sites along the genome but are not themselves sufficiently close to yield 5S-45S Hi-C contacts. Ribosomal DNA contacts are enriched in segments of closed, repressed, and late replicating chromatin, as well as CTCF binding sites. Finally, we identified functional categories whose dispersed genes coalesced in proximity to the rDNA arrays or instead avoided proximity with the rDNA arrays. The observations further our understanding of the spatial localization of rDNA arrays and their contribution to the architecture of the cell nucleus.

## Author summary

The repeated ribosomal DNA (rDNA) array gives rise to the nucleolus, an organelle that is involved in key cellular processes such as stress response, cell cycle regulation, RNA modification, and production of more than 70% of all cellular RNAs (the ribosomal RNAs). This critical genome element has remained unassembled and has been excluded from all Hi-C analyses to date. Here we built the first map of 5S and 45S rDNA contacts with the rest of the genome. The map yielded a number of novel results and challenge the expectation that 5S and 45S arrays are close together in the nucleus. The rDNA arrays share common sites of contact across the genome, are biased towards segments of closed, repressed, and late replicating chromatin, and display greater proximity or avoidance to functionally coherent gene sets. The results further our understanding of the rDNA arrays and their localization in the nuclear environment.

## Introduction

Ribosomal RNAs (rRNAs) are essential components of the cell, and are encoded in the 5S and 45S ribosomal DNA (rDNA) arrays of higher eukaryotes [1–4]. The 5S rDNA array resides on chromosome 1 and encodes the 5S rRNA, whereas the 45S rDNA array resides on five human acrocentric chromosomes and encodes the 18S, 5.8S, and 28S rRNA components of the ribosome [5–7]. The nucleolus, the first recognized nuclear organelle, is the site of 45S rRNA transcription [1, 2, 4, 8]. The lack of homology between the 5S rDNA and the subunits of the 45S rDNAs arrays reflect deep evolutionary separation. For instance, RNA polymerase I is exclusively dedicated to the transcription of the 45S rRNA, while RNA polymerase III transcribes the 5S rRNAs and tRNAs. The distinct RNA polymerase machineries required for transcription of 5S and 45S subunits are a conserved feature of yeasts, plants, fruit flies, and humans. Furthermore, distance to the nucleolus is thought to be relevant for global gene expression. For instance, proximity to the nucleolus can in some cases promote inactivation of certain RNA polymerase II transcribed genes [9], although the observation has not been systematically tested across the genome. Finally, localization of the 5S array has been documented at the periphery of the nucleolus [9, 10], but also away from the organelle [11], with a substantial fraction of cells showing 5S arrays that are localized elsewhere in the nucleus [10]. Uncovering physical contacts between the rDNA arrays and the rest of the genome can expand our understanding of nuclear architecture, nucleolar structure and function, and the mechanism of concerted copy number variation between 5S and 45S rDNA arrays. However, studies of nuclear architecture have largely excluded analyses of spatial interactions with the 5S and 45S rDNA arrays.

Ligation-capture Hi-C sequencing technology [12–14] enabled a revolution in our understanding of nuclear organization with the identification of hundreds of topologically associated domains (TADs). Human TADs span an average 900 KB each and display remarkable conservation with TADs identified in mice. TADs display, moreover, remarkable structural stability through development and when cells are perturbed in gene knockdown experiments [15, 16]. On the other hand, deep sequencing of nucleoli led to the documentation of nucleoli associated DNA (naDNA) and the identification of nucleolus associated domains (NADs) [17–19]. While NADs display size variation spanning multiple orders of magnitude, they are generally large. NADs covering less than 0.1 MB are relatively rare with most NADs around 1 MB or larger. The domains encompass about 5% of the human genome, are represented in all chromosomes, and are now recognized to be stably associated with nucleoli. Analysis of rDNA interactions with Hi-C might provide a complementary approach to localize the rDNA in the nuclear space possibly informing nucleolar interactions with the genome at a different scale than those afforded by analysis of naDNA.

Here we addressed the landscape of long-range rDNA interactions with 16,482,743 reads identified from a total of >15 billion (15,165,355,427) Hi-C reads in five cell types and two cell lines. The data enabled a map of long-range rDNA interactions at 1MB resolution, and the identification of segments displaying statistically significant differential contact density between cells. The map yielded a number of observations and suggest that the 5S and 45S arrays are not as spatially close as typically expected, yet share significant overlap with common contacts elsewhere. Finally, the data uncovered functionally coherent categories whose dispersed genes either coalesce in proximity to the rDNA arrays or avoid proximity with the rDNA arrays.

## Results

### Ribosomal DNA containing reads in Hi-C

We investigated human Hi-C data for two cell lines and five cell types; the two cell lines represent the most replicated human Hi-C datasets to date, yet yielded a relatively small number of rDNA informative reads. For instance, we mined 5,356,990,189 high quality Hi-C reads in LCL to identify 13,528,436 reads with at least one end mapped to the 45S rDNA and 105,147 reads with at least one end mapped to the 5S rDNA (S1 and S2 Tables). Similarly, for K562 cells, we mined 903,837,936 high quality Hi-C reads to identify 1,698,063 reads with at least one end mapped to the 45S rDNA and 47,691 reads with at least one end mapped to the 5S rDNA. This represents a 0.25% and 0.19% recovery rate of 45S rDNA reads in shotgun Hi-C in LCL and K562, respectively. These numbers were substantially larger than the meager 0.002% and 0.005% recovery rate for 5S rDNA reads in LCL and K562, respectively. Similar recovery rates were obtained with the other five cell types studied (Table 1). Overall, we uncovered 16,322,538 reads with at least one end mapped to the 45S rDNA and 160,205 reads with at least one end mapped to the 5S rDNA (Table 1). The mining effort illustrates the challenge in recovering rDNA information in shotgun Hi-C experiments. Nevertheless, the data revealed that rDNA contacts are dispersed across the entire genome, with segments differing in the density of rDNA interaction. The maps also revealed that naDNA and rDNA-contacts are not overlapping domains and likely reflect different attributes of the nucleolus/rDNA (S1 Fig).

**Table 1:** Summary table for the analyzed Hi-C reads from two cell lines and five cell types. Shown are the number of reads analyzed per set and the number of reads remaining after the quality control (QC) steps. Shown are the count and percentage of QC reads that mapped to the rDNA arrays. Data is shown for both 5S and 45S rDNA arrays.

Datasets	Number of reads	Number of reads after trimming and QC	% kept after QC	45s reads	% of 45S reads	5s reads	% of 5S reads
ESC	1,965,460,504	293,548,127	14.94	300,903	0.10	1,627	0.0006
Mesod	1,453,457,218	282,703,604	19.45	160,118	0.06	1,217	0.0004
Mesec	1,946,805,860	375,059,953	19.27	167,365	0.04	765	0.0002
Neuro	1,478,607,926	284,198,790	19.22	287,756	0.10	1,449	0.0005
Ectod	936,917,416	181,739,870	19.40	179,897	0.10	2,309	0.0013
K562	1,366,228,845	903,837,936	66.16	1,698,063	0.19	47,691	0.0053
LCL	6,017,877,658	5,356,990,189	89.02	13,528,436	0.25	105,147	0.0020
SUM	15,165,355,427	7,678,078,469	51.63	16,322,538	0.21	160,205	0.0020

### Ribosomal DNA contact maps at 1MB resolution

**Fig 1:**
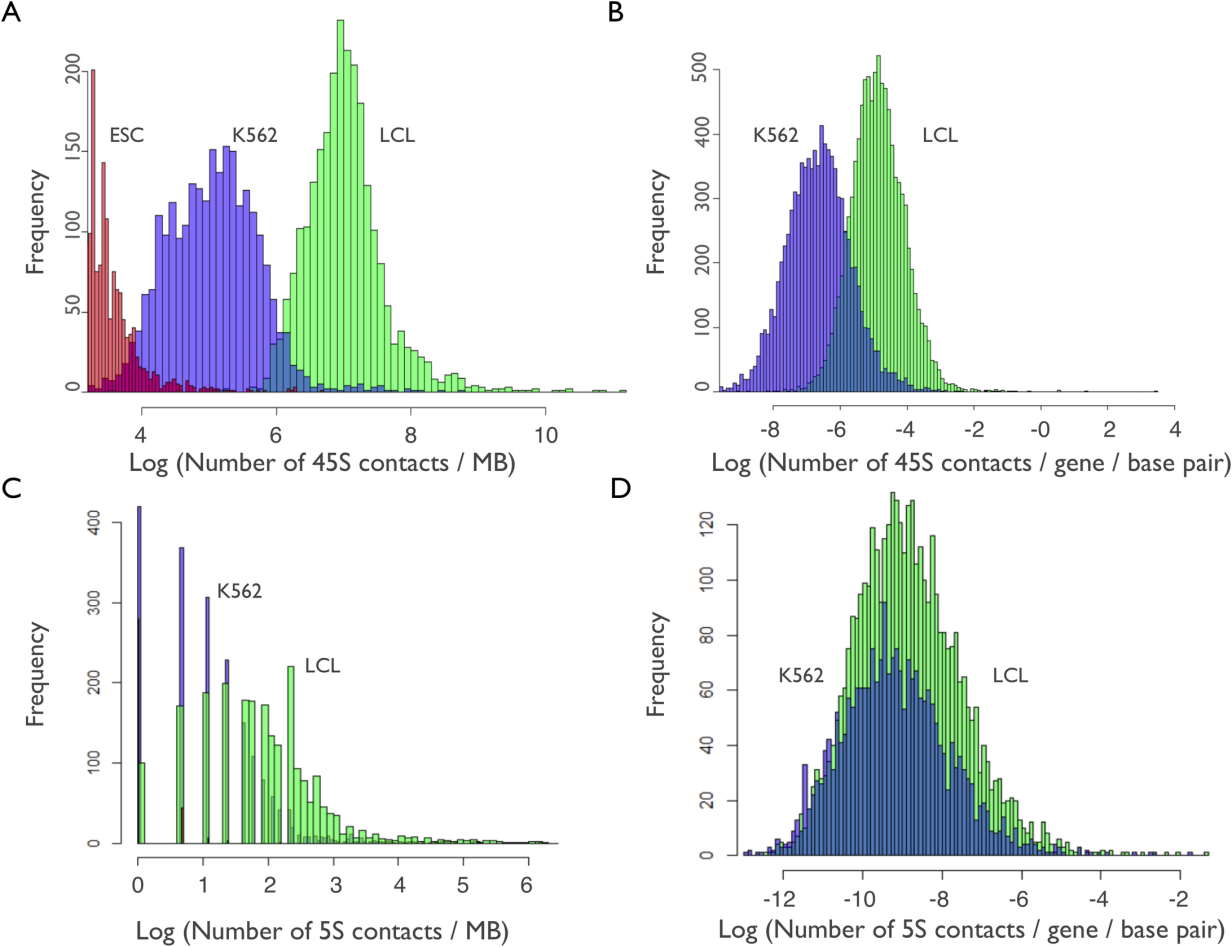
Extensive variation in ribosomal DNA (rDNA) contacts across protein-coding genes and 1MB genomic segments. (A) Distribution of the number of 45S contacts (log scale) in 1MB genomic segments for LCL (green), K562 (purple) and ESC (red). (B) Distribution of the number of 45S rDNA-gene contacts per base pair (log scale) in LCL and K562. (C) Distribution of the number of 5S contacts (log scale) in 1MB segments for LCL and K562. (D) Distribution of the number of 5S rDNA-gene contacts per base pair (log scale) in LCL and K562. Only segments and genes without rDNA pseudogenes are included.

Here we partitioned human autosomes (Chr 1 to 22) into 2897 segments of 1MB, 2465 and 2658 of which had no evidence of containing a 5S or 45S pseudogene, respectively. Segments containing an rDNA pseudogene were disproportionately found adjacent to centromeric and telomeric regions, and were excluded from all further analyzes. Unsurprisingly, all 1MB segments across all chromosomes displayed evidence of rDNA contact (S1 and S2 Figs). Moreover, at the 1MB scale, we observed good reproducibility between replicates of a cell line using the same restriction enzyme as well as different restriction enzymes, with consistent results across biological replicates and across cell lines/cell types (S3, S4, S5, S6, S7, S8 and S9 Figs). Fig 1 illustrates the distribution of rDNA contact density for 1MB segments before normalization by sequencing effort. The data shows a 5-10-fold variation in the logarithm of the contact density across segments within a cell type. The mean difference in the average contact density among cells reflects variation in the amount of Hi-C data in each cell type. For 45S rDNA contacts all 1MB segments contained appreciable density of contacts in LCL and K562. However, the ESC and ESC-derived cell types (ESC set) displayed a truncated distribution with many segments that contained very few rDNA contacts (Fig 1A). This was due to the lower number of Hi-C reads for those cells (Table 1). The resolution was much worse for the 5S rDNA arrays (Fig 1C). Therefore, the following analyses focused primarily in the data for LCL and K562 cell lines, with the ESC or ESC-derived cells mostly used for comparisons.

### Differential ribosomal DNA contacts density at 1MB resolution

**Fig 2:**
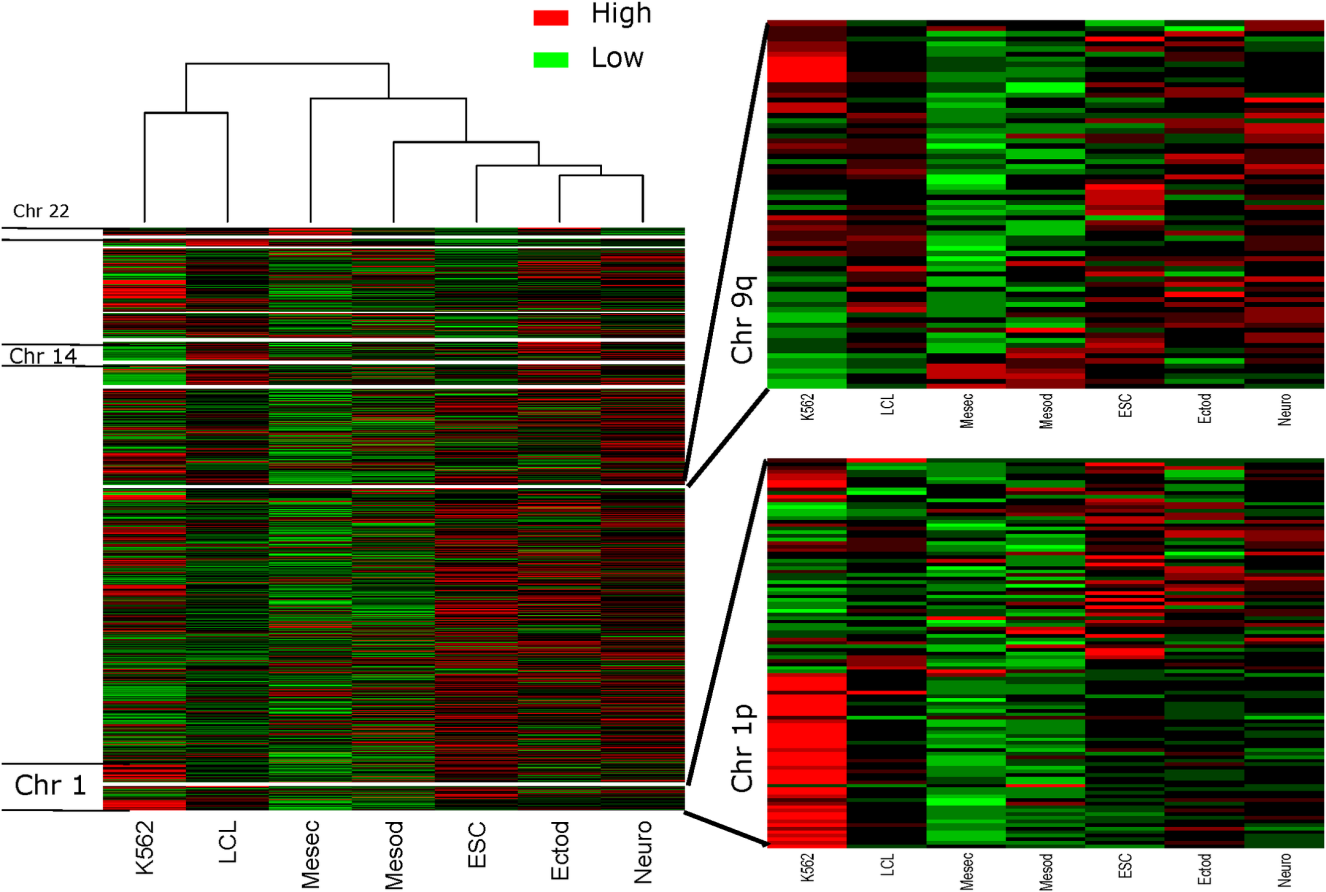
Diversity of 45S rDNA contact density in 1MB segments across seven datasets (five cell types and two cell lines). Heatmaps display data for the whole genome (Left panel), a region of Chromosome 9 (Right upper panel), and a region of chromosome 16 (Right lower panel). The number of reads in each segment is normalized by the total number of rDNA reads in each cell line or cell type. Statistically significant differences in rDNA contact density are described in the text and were assessed separately for the LCL vs. K562 contrast and across the five ESC derived cell types.

**Fig 3:**
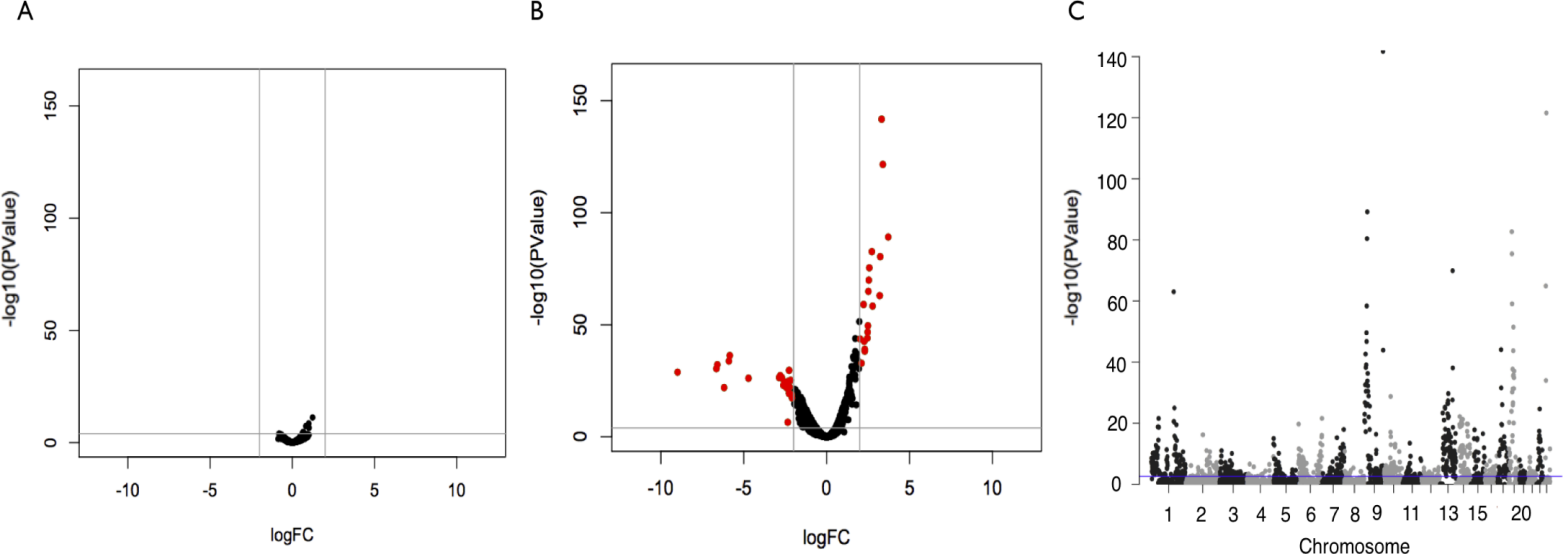
Ascertaining significant 45S rDNA contact density variation in 1MB segments between LCL and K562 cells. (A) Volcano plot for the contrast between biological replicates of LCL collected with different enzymes (Mbol and DpnII). (B) Volcano plot for the contrast between LCL vs K562 replicates collected with the same enzyme (Mbol). Red dots represent segments with difference larger than two-fold change (vertical line) and P-value < 3 × 10^−4^. (horizontal line). (C) Manhattan plot showing chromosome distribution of P-values for the contrasts between LCL and K562. The X-axis shows chromosomal position. The Y-axis shows −log10 (P). The blue line denotes a P = 3 × 10^−4^.

Here we first addressed variation in rDNA contact density across cell lines (LCL vs K562 data collected with the same enzyme and protocol). We found 808 segments of 1MB with significantly different Density of Interactions (DI) of 45S rDNA contacts between LCL and K562 (Fig 2; Fig 3, FDR < 0.05; S3 Table), whereas none is identified among biological replicates of LCLs processed with different enzymes (Fig 3). We observed that 350 DI segments displayed increased density in LCL and 458 segments displayed increased density in K562. Among those 808 DI segments, 302 of them displayed a greater than 2-fold difference in contact density between LCL vs K562. Similarly, nearly half of the 224 segments of 1MB in chromosome 1 showed evidence of DI density between LCL and K562 (Fig 3; chromosome 1: 106 segments significantly different, and 118 non-significant bins), with 97 segments displaying greater contact density in LCL and 9 segments containing greater contact density in K562. Chromosome 1 had the largest number of significantly different DI, followed by Chr 13 (89), Chr 9 (63), and Chr 6 (61). Among the five cell types (ESC related), there were 193 segments of 1MB with significantly different DI with the rDNA (FDR<0.05; S4 Table). Finally, we detected a meager 15 segments with evidence of differential DI between LCL and K562 for the 5S rDNA (FDR<0.05); the small number of differential DI likely reflects the many fewer 5S rDNA reads and thus the much-lowered statistical power of this analysis. Similarly, there was not enough Hi-C data to enable statistical analysis of DI with the 5S rDNA among the five ESC related cell types.

### Identification of rDNA-gene contacts

We identified 9,595 and 9,864 genes without evidence of a 5S or 45S rDNA pseudogene, respectively. The remaining genes were excluded from all further analyzes. The data showed a continuous distribution of rDNA-gene contact density for the 45S and 5S rDNA (Fig 1B), with much better resolution for the 45S rDNA than for the 5S rDNA (Table 2, Table 3). As expected, the rDNA-gene contact density was correlated with gene length. We have thus calculated the 45S contact density per gene per nucleotide (“Contacts per gene per nucleotide, CPGN”). This removed the correlations between gene length and 45S rDNA contacts and revealed that CPGN for the 45S rDNA arrays was strongly correlated between LCL and K562 (rho = 0.65; P < 0.001). This correlation was stronger than those between LCL and ESC (rho = 0.27; P < 0.001) or between K562 and ESC (rho = 0.34; P < 0.001). The lower correlations with ESC might partially reflect the lower resolution of the ESC contact map with a substantial fraction of genes showing less than 10 reads with rDNA contacts (Table 2). Indeed, although the overall amount of HI-C data was large, the resolution to ascertain 45S rDNA-gene contacts was only sufficient for LCL and K562, the two biological sources with the largest number of Hi-C reads to date. The issue of low rDNA-gene resolution was particularly evident for the 5S rDNA. Out of 9595 genes analyzed for 45S rDNA arrays, there were 67 and 612 genes with zero 5S contacts in LCL and K562, respectively. For the ESC set, however, there were 1745 genes with zero contacts with the 45S rDNA arrays. Out of 9864 genes analyzed for 5S rDNA arrays, there were 5916 and 7494 genes with zero 5S contacts in LCL and K562, respectively. For the ESC set, we observed that greater than 95% of the genes had zero 5S contacts. The density of 5S rDNA-gene contacts was most strongly correlated with gene length (rho > 0.3, P < 0.001), but calculating the 5S contact density per gene per nucleotide (“Contacts per gene, CPGN”) removed the positive association. Among genes with at least one read showing 5S-rDNA contact in both LCL and K562 we found that CPGN is strongly correlated between LCL and K562 (rho = 0.64, P < 0.001). Evidence for a positive association between the density of 45S rDNA contacts and the density of 5S rDNA contacts is also observed in other partitions of the data, and across genes and 1MB segments in both LCL and K562 (Table 4).

**Table 2:** Summary table for the number of genes with 45S rDNA contacts. Shown are the number of genes with exactly 0 contacts, with >1 and <10 contacts, and with >10 contacts for each cell type and cell line.

Count 45S	ESC	Mesod	Mesec	Neuro	Ectod	K562	LCL
0	6271	3862	2799	3275	3118	662	89
>1 & <10	1690	2073	1821	1509	1190	910	150
>10	1824	3850	5165	5001	5477	8213	9546

**Table 3:** Summary table for the number of genes with 45S rDNA contacts. Shown are the number of genes with exactly 0 contacts, with >1 and <10 contacts, and with >10 contacts for each cell type and cell line.

Count 5S	ESC	Mesod	Mesec	Neuro	Ectod	K562	LCL
0	9637	9692	9684	9689	9726	7449	5886
>1 & <10	111	65	65	67	44	1395	2022
>10	37	28	36	29	15	941	1877

**Table 4:** Summary table for the spearman rank correlations between 45S and 5S rDNA contacts. The correlation was computed across genes and across non-overlapping 1MB segments in LCL and K562 cells. All correlations are statistically significant (P < 2 x 10^−16^, in all cases).

	All genes	Genes with more than 1 contact with both 5S and 45S	Genes with more than 10 contacts with both 5S and 45S
LCL	ρ = 0.42	ρ = 0.41	ρ = 0.18
K562	ρ = 0.35	ρ = 0.37	ρ = 0.13
	**All 1MB segments**	**1MB segments with more than 1 contact with 5S**	
LCL	ρ = 0.55	ρ = 0.42	
K562	ρ = 0.60	ρ = 0.52	

### Differential rDNA-gene contact density

Here we tested for variation in rDNA-gene contact density between LCL and K562. For the 45S array, we observed 731 genes with fold change in interaction density >2 for the LCL vs K562 comparison (experiments with the same enzyme and protocol); 97 genes (FDR < 0.05) displayed significantly different DI after multiple corrections (S10 Fig). For the analyses of 45S rDNA contacts variation among five ESC related cell types, we observed 435 genes with significantly differential density of rDNA contacts (FDR < 0.05). For the 5S array, we observed 954 genes with DI fold change >2 in the LCL vs. K562 comparison. However, none of these genes reached statistical significance, possibly due to the higher variance emerging from the low coverage and thus limited number of 5S contacts in each gene. There was not enough data for statistical analyses of variation in 5S rDNA contact among the five ESC related cell types.

### Per nucleotide rDNA contact rates

Here we estimated contact densities per base pair in three ways. First, the average contact per base pair across the whole genome was calculated by dividing the total number of mapped rDNA-genome reads by the genome length (3 billion base pairs). The average contact rate is estimated as 4.8 x 10^−5^ and 3.7 x 10^−3^ contacts per base pair for the 5S and 45S rDNA, respectively (S5 Table). Hence, for the 45S rDNA each base pair in the genome is expected to have 0.37 mapped reads. Second, the average contact per base pair was estimated after filtering out bins with pseudogenes. Here we divided the total number of rDNA-genome reads within 1MB segments without a pseudogene by the total sequence length in those segments. This yielded an estimated average contact rate of 2.0 x 10^−5^ and 1.7 x 10^−3^ contacts per base pair for the 5S and 45S rDNA, respectively. These numbers are comparable with those estimates using all rDNA reads and the whole genome. Third, we estimated the average contact rate per base pair in protein-coding genes by dividing the total number of rDNA-gene reads by the total length of nucleotides within genes, after excluding genes with evidence of containing rDNA pseudogenes. This yielded an average contact rate for genic segments of 2.2 x 10^−4^ and 0.016 contacts per base pair for the 5S and 45S rDNA, respectively (S5 Table). Collectively, these estimates of contact rate are useful in evaluating regions with putative enrichment or deficit in rDNA contacts.

### rDNA contacts preferentially occur in close, repressed, late replicating domains

**Fig 4:**
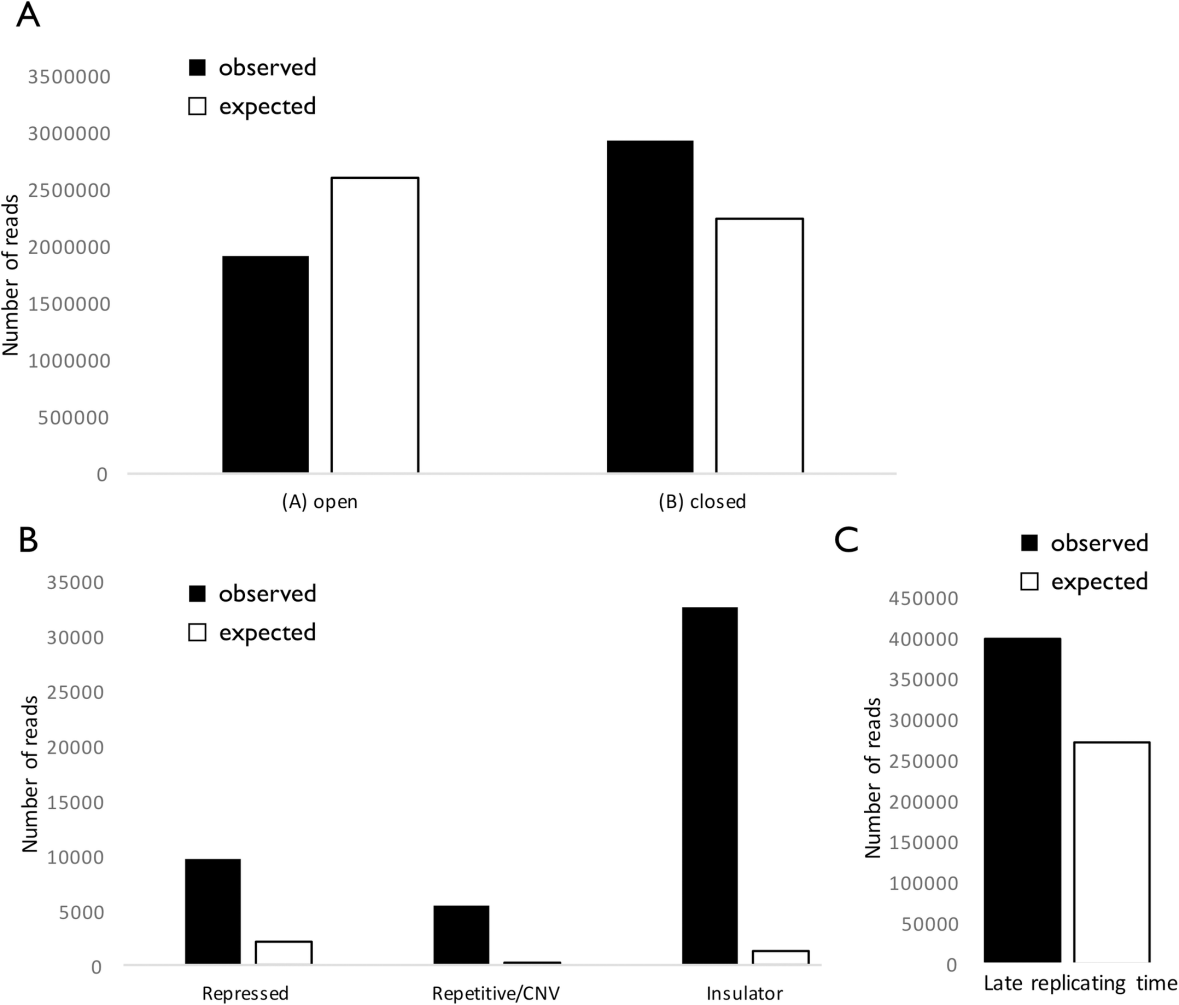
Closed, repressed, late replicating chromatin segments are preferentially associated with the rDNA array. The number of observed (black) and expected (white) rDNA contacts with each functional annotation is shown. Expected numbers are calculated with the genome-wide per nucleotide contact rate. Data for (A) open A / closed B compartments in LCLs as computed in [60 106]; (B) three selected annotations from 15-label genomic segmentation of hESC using ChromHMM [61]; (C) the 5% slowest replicating segments of the genome as recorded in the Replication Domain Database.

We examined the relationship between various genomic attributes and the density of rDNA contacts. First, the data showed a significant association between the number of 45S rDNA contacts and the A/B compartments. Specifically, the B compartment of closed chromatin displays an enrichment in rDNA contacts, whereas the A compartment of open chromatin displays a deficit of rDNA contacts (P < 0.01, Chi-square test; Fig 4). In addition, we examined 15 functional annotations; significant enrichments were observed in segments of repressive chromatin, as well as in segments annotated as repetitive or containing insulator regions (P < 0.01, Chi-square test; Fig 4; S11 Fig). Finally, we examined segments of CTCF binding; CTCF is a conserved 11-zinc finger DNA binding protein that regulates chromosome architecture [20]. Using the CTCF database we estimated that CTCF binding segments constitute <7.5% of the human genome. On the other hand, we observed that 37% and 29% of all 45S rDNA-genome reads overlapped a CTCF binding segment in LCL and K562, respectively. These figures are in good agreement with the 35% of all rDNA-genome reads that overlapped a CTCF binding segment in the ESC cell set. These represent a >4-fold enrichment that indicate a significantly higher percentage of 45S rDNA contacts with CTCF binding sites (P < 0.05, one proportion test).

### Genes associated with rDNA CN variation are not enriched in rDNA contacts

**Fig 5:**
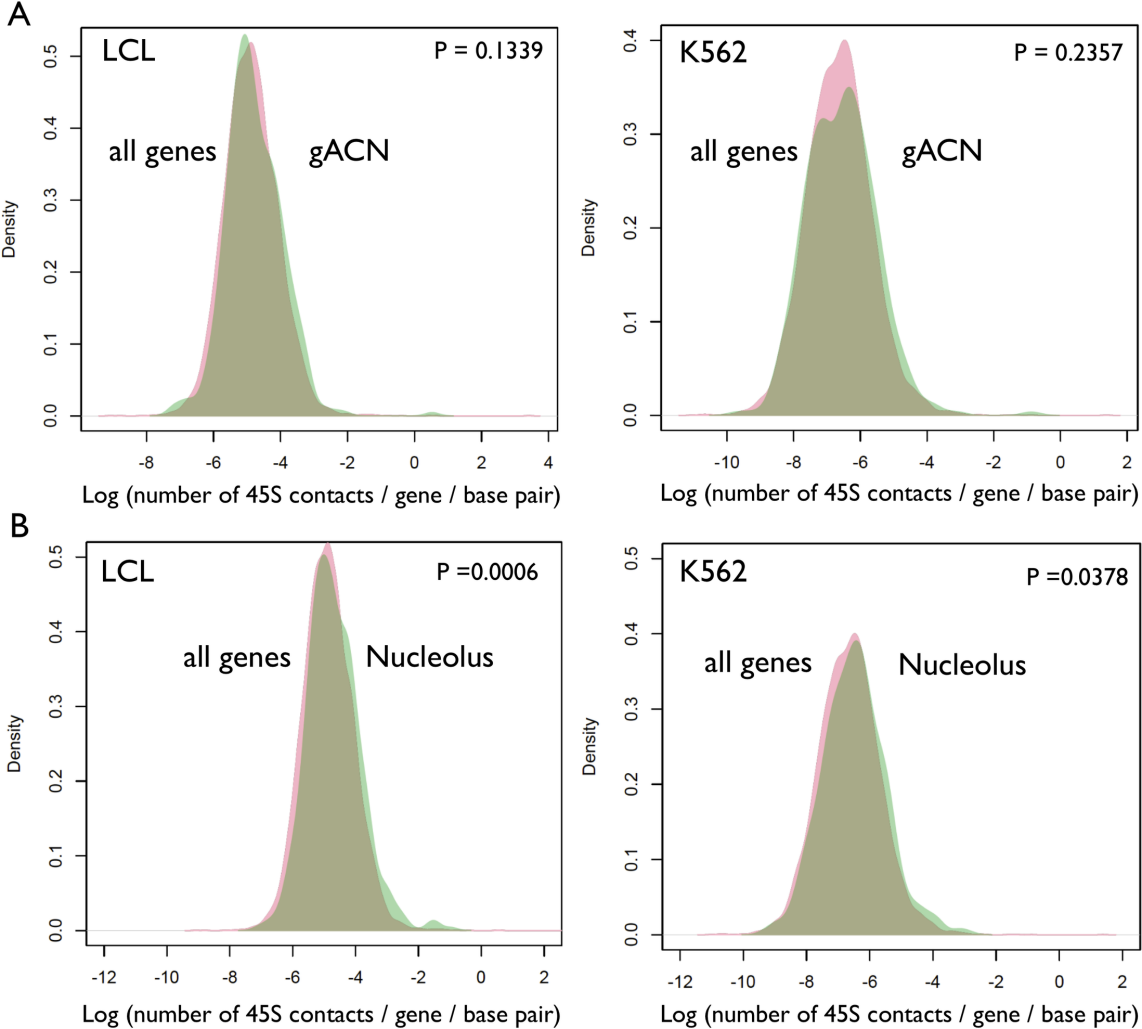
Distribution of 45S-gene contacts across genes belonging to selected functional categories in LCL and K562. (A) genes whose expression is Associated with rDNA Copy Number variation (gACN, green color). Associations from [21]. (B) genes localized to the nucleolus (green color). The red colored densities display the distribution of rDNA-gene contacts across all genes in LCL or K562. Statistical significance was assessed with Kolmogorov-Smirnov tests.

**Fig 6:**
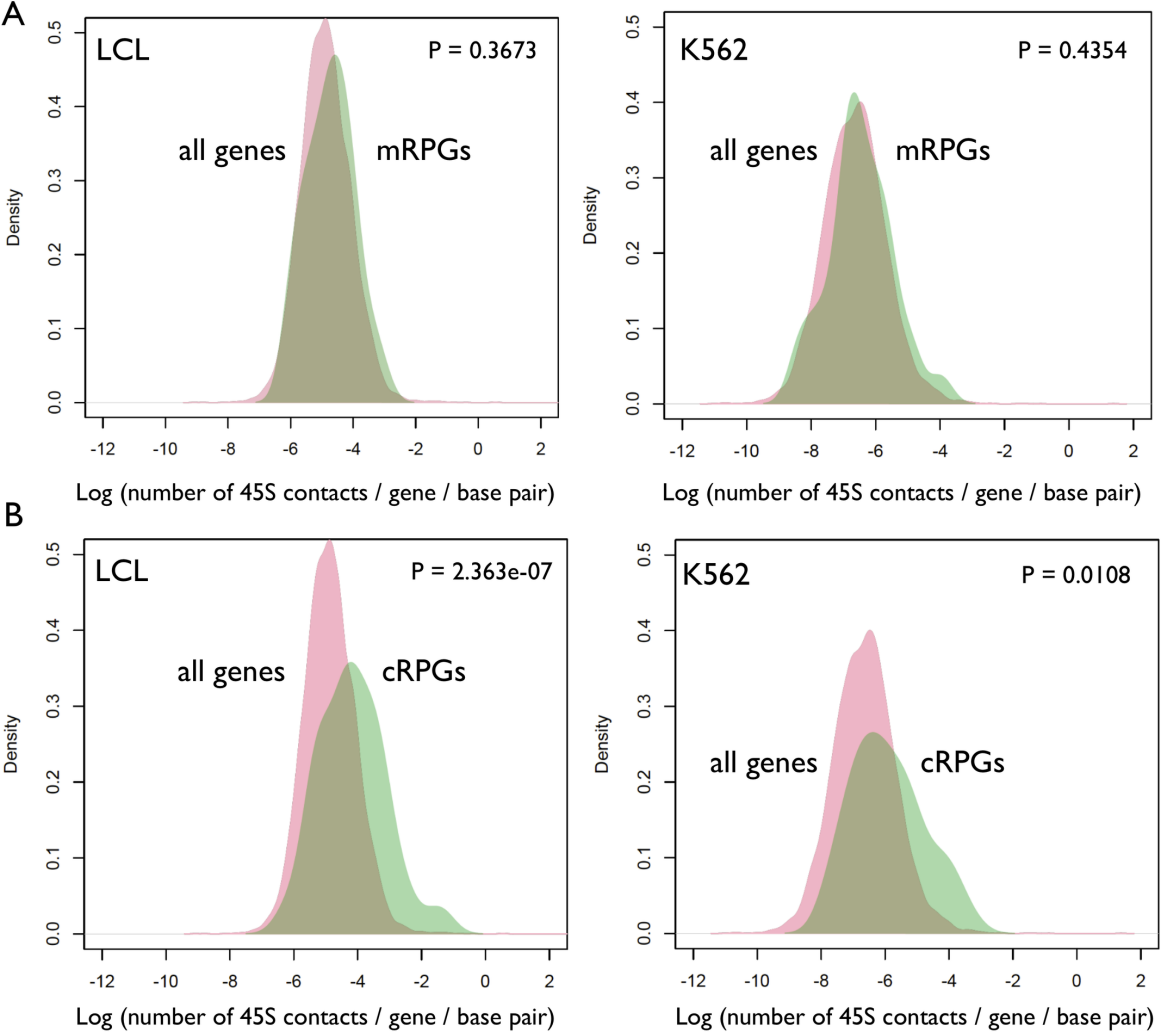
Distribution of 45S-gene contacts across genes encoding the protein components of the ribosome in LCL and K562. (A) Genes encoding protein components of the mitochondrial ribosome (mRPGs, green color). (B) genes encoding protein components of the cytosolic ribosome (cRPGs, green color). The red colored densities display the distribution of rDNA-gene contacts across all genes in LCL or K562. Statistical significance was assessed with Kolmogorov-Smirnov tests.

We selected a small set of genes to be examined in greater detail. Specifically, we examined genes that are (i) known to regulate rDNA function or structure and/or (ii) whose expression are associated with rDNA CN variation [21–23]. For instance, the *CTCF* gene is located on Chr16 and displayed a meager 118 contacts with the 45S rDNA in LCLs, which is significantly lower (P-value < 0.001, one proportion test) than the expected 1198 contacts calculated based on the genome wide average contacts per base pair (1.56%) and the length of the *CTCF* gene. Thus, the *CTCF* gene appears to be in repulsion to the rDNA arrays. Similarly, *CBX1*(*Hp1beta*), *Ubf1*, and *KDM4B* had fewer hits than expected (P < 0.0001 for all of them, one proportion test). Thus, we examined the top 400 genes that are positively and negatively associated with rDNA CN variation in LCL [21]. Collectively, however, these genes were neither enriched nor depleted in rDNA contacts, with a distribution of contacts that is undistinguishable from all other genes in the genome (Fig 5). Nevertheless, nucleolar, mitochondrial, and ribosomal genes were also associated with variation in rDNA array CN [21], and could reveal a distinct pattern. Accordingly, genes that localize to the nucleolus as well as ribosomal genes showed a distribution of contacts that was significantly shifted towards a greater than average number of contacts with the rDNA array in both LCLs and K562 (Fig 5 and Fig 6).

### Ribosomal and mitochondria-related genes are enriched in rDNA contacts

Next, we addressed if the higher density of rDNA contacts in nucleolar, ribosomal, and mitochondrial genes would emerge as significant gene ontology enrichments when genes with a high CPGN are selected. To address the issue, we examined the genes in the top 5% higher number of 5S and 45S contacts after correction for gene length (i.e., CPGN). For 5S rDNA-gene contacts in LCL the cell component category of mitochondrion (GO:0005739) emerged on the top of the list, with 56 candidates (out of 494 genes) localized to the mitochondrion. The association is functionally intriguing and also emerged in the K562 dataset (S6 Table). The same class emerged among the top 5% in the 45S rDNA in LCL, with 63 candidates in the mitochondrion (GO:0005739; adjusted P < 0.05, after correction for multiple testing). The class includes interesting candidates such as seryl-tRNA synthetase 2 (mitochondrial SARS2; ENSG00000104835), tRNA 5-methylaminomethyl-2-thiouridylate methyltransferase (TRMU; ENSG00000100416) and tRNA methyltransferase 1 (TRMT1; ENSG00000104907), Era like 12S mitochondrial rRNA chaperone 1 (ERAL1; ENSG00000132591). In addition, 10 other functionally coherent cell components emerged for 45S rDNA-gene contacts in LCL (S7 Table; adjusted P < 0.05, for all classes in LCL; see S8 and S9 Tables for data on K562 and the ESC set). Four of those categories are highly significant GO terms containing the protein-components of the ribosome (GO:0005840~ribosome, GO:0022625~cytosolic large ribosomal subunit, GO:0015935~small ribosomal subunit, and GO:0022627~cytosolic small ribosomal subunit). Collectively, the data suggest that highly transcribed genes encoding protein constituents of the ribosome are co-localized in proximity to the rDNA arrays (Table 5). In addition, one GO term related to nucleolar function (GO:0005730~nucleolus) also emerged as significantly enriched with 39 genes in the top 5% of genes with higher numbers of 45S rDNA-gene contacts in LCL. Genes in this set include intriguing candidates such as NOP2 nucleolar protein (NOP2; ENSG00000111641), FSHD region gene 1 (FRG1; ENSG00000109536), Sirtuin 6 (SIRT6; ENSG00000077463), and MDM2 (ENSG00000135679).

**Table 5:** Summary table for gene ontology (GO) enrichments for genes in the top 5% higher number of 45S rDNA contacts in LCLs, K562, and ESC derived cell types. Shown are the number of genes represented in each GO category. Raw and adjusted P-values are listed. Adjusted P-values (in brackets) were obtained after Benjamini-Hochberg correction. “ESC set” refers to the sum of contacts in ESC and the four ESC derived cell types.

GO term	GO ID	LCL Gene count	LCL P-value (adjusted)	K562 Gene count	K562 P-value (adjusted)	ESC set Gene count	ESC set P-value (adjusted)
ribosome	GO:0005840	18	9E-07 (0.00013)	13	0.001 (0.067)	9	0.058 (0.66)
mitochondrion	GO:0005739	63	1.3E-06 (0.00014)	50	0.004 (0.17)	45	0.035 (0.60)
cytosolic large ribosomal subunit	GO:0022625	10	0.00005 (0.0039)	6	0.027 (0.56)	7	0.007 (0.29)
spliceosomal complex	GO:0005681	11	0.00012 (0.008)	6	0.085 (0.78)	8	0.009 (0.31)
nucleolus	GO:0005730	39	0.00044 (0.021)	31	0.038 (0.64)	36	0.003 (0.16)

### Developmental genes are depleted in rDNA contacts

Among genes in the bottom 5% CPGN in 45S, we observed seven HOX genes dispersed across several chromosomes (HOXA1, HOXA6, HOXA7, and HOXA11 on Chr 7, HOXB5 on Chr 17, HOXC11 on Chr 12, and HOXD13 on Chr 2), three of which showed zero 45S rDNA contacts [HOXA7(Chr 7), HOXC11(Chr 12), and HOXD13(Chr 2)] even in the dense LCL map. This suggests that developmentally regulated Hox genes are rarely localized in proximity to the rDNA arrays. Furthermore, we also found several other developmental genes in the set of 67 genes with zero contacts with 45S rDNA genes, further indicating that developmental genes show “repulsion” from the rDNA genes. Interesting candidates include NK2 homeobox 3 (NKX2-3) on Chr 10, BMP3 on Chr 4, BMP5 and BMP6 on Chr 6, as well as NOTCH1 on Chr 9. Interestingly, the histone cluster 1 H1 family member d (HIST1H1D) on Chr 6 also emerged without a single 45S rDNA contact in the dense 45S map of LCLs. Finally, we confirmed the lack of Hi-C contacts between the 5S and 45S arrays [24]. The segments proximal to the 5S array also displayed depletion in 45S rDNA contacts. The gene RHOU, for instance, is located adjacent to the 5S array and emerged in the bottom 3% of the distribution of 45S rDNA contact density.

## Discussion

Multicopy ribosomal DNA arrays are essential components of the genome. Yet ribosomal DNA arrays are also among the most variable segments of the genome. The arrays have lagged behind with limited assemblies and little understanding of their nuclear localization. Here we report a detailed contact map of spatial interactions between the rDNA arrays and the rest of the genome. Although there are huge amounts of HI-C data, analyses of rDNA contact density for specific regions/genes remained a challenge because rDNA reads constitute a fraction of the Hi-C reads. Thus, we combined multiple Hi-C datasets to identify the subset of reads containing information on rDNA contacts. The effort was computational intensive because the fraction of rDNA reads in shotgun Hi-C is very small. This is particularly evident in the case of the 5S rDNA array: the contact data remained sparse even for LCL, the cell line that has by far the largest amounts of data collected from multiple Hi-C experiments. Nevertheless, we identified consistency of rDNA-gene contacts across different cells (LCL and K562; especially for 45S), which point to replicable spatial interactions. Heatmaps enabled visualization of rDNA contacts along the human genome with statistical analyses pinpointing significant differences in the density of contacts. While the approach can be applied to other multicopy genes as well as single copy genes or regions, we caution that the typical resolution of shotgun Hi-C is not sufficiently high. Indeed, limited resolution was apparent for both the 5S rDNA and 45S rDNA arrays, which required combining multiple datasets to ascertain contacts with genic and non-genic segments of the genome. In summary, the LCL map achieved good resolution for 5S and 45S contacts but the K562 set is quite sparse for 5S contacts, and both 5S and 45S maps are very sparse in the case of the ESC cell and ESC-derived cell types.

Variation in rDNA contact density across genes reflects variation in proximity to the rDNA arrays. The data displayed over 100-fold variation in contact density across genes and revealed several intriguing patterns. First, the compilation enabled us to conduct statistical tests of the differential density of rDNA interactions between LCL and K562. These 45S maps are sufficiently dense, with differences in contact density likely reflecting differences in nuclear organization between these cells. These differences are not surprising since the LCLs are immortalized cells derived from lymphocytes whereas K562 is a myelogenous leukemia. K562 has, moreover, undergone genomic rearrangements [25]. While the data also suggested variation across ESC and ESC-derived cell types, greater coverage for these cells is necessary to draw sufficiently dense contact maps for a more fine-grained and meaningful biological contrast. An intriguing suggestion is that the rDNA/nucleolus represents a keystone in nuclear structure around which the rest of the genome is functionally organized [26] [24]. In this case, rDNA-contact differences between cells are bound to emerge and reflect functional variation.

Second, as a class, the rDNA proximity with genes previously identified as associated with rDNA CN variation across genotypes in human populations is undistinguishable from the background of genes. This indicates that genes impacted by rDNA CN are not spatially close to the rDNA arrays and are not enriched in direct rDNA contacts. This is not an unexpected observation, because the association of gene expression variation with rDNA CN includes hundreds of genes, with only a fraction of them likely to be directly regulated by the rDNA array (i.e. genes associated with rDNA CN are presumably modulated by both direct and indirect effects emerging from the rDNA). While we suggest that changes in nuclear architecture could be one way to for the rDNA to exert regulatory effects, the mechanisms through which rDNA CN directly modulates gene expression are likely varied and the ratio of direct to indirect effects is unknown. Third, we observed that genes encoding proteins that localize to the mitochondria display a disproportionally large number of contacts with the 45S rDNA. Concordantly, genes localized to the mitochondria also emerged as enriched in 5S rDNA contacts. The data suggests that genes localized to the mitochondria might be collectively regulated through aspects of nuclear architecture that are influenced by the rDNA. Noteworthy, connections between the rDNA array and mitochondrial gene expression and function have been uncovered before. In Drosophila, Paredes et al (2011) observed that engineered deletions in the rDNA array preferentially impacted the expression of genes whose protein products localized to the mitochondrion [27]. In humans, Gibbons et al (2014) observed that rDNA CN variation is associated with the expression of genes whose protein product localize to the mitochondrion as well as genes encoding protein components of the mitochondrial ribosome and mitochondrial DNA copy number [21]. Interestingly, in addition to its well-documented role as a structural component of the cytosolic ribosome, the 5S rRNA is also specifically imported into the mitochondria [28, 29].

Fourth, we observed that genes localized to the nucleolus and encoding protein components of the ribosome were significantly enriched for 45S rDNA contacts. The finding points to the specificity of rDNA-genome interactions and suggests that ribosomal gene regulation might be directly influenced by the rDNA array. This pattern of rDNA-gene contacts might partially explain the observation that genes whose expression was correlated with rDNA CN included several candidates encoding the protein components of the ribosome. Indeed, sequence specific inter-chromosomal interactions between the yeast rDNA array and an intergenic segment adjacent to the largest RNA pol I subunit has recently been demonstrated [30]. All in all, our study identified functionally coherent genes and GO categories that are depleted and enriched in direct rDNA contacts. Ribosomal DNA contacted regions for all chromosomes along the human genome suggest a structural component underlying the global regulatory consequence of rDNA CN variation [21]. Finally, we note that as much as 29% of the 45S rDNA reads have both ends mapped in the 45S rDNA. These partially reflect linear proximity along the 45S rDNA unit but could also emerge from looping substructures with contacts between distant units; looping and contact among non-adjacent units has been suggested to facilitate ultra-structural organization of the array and coordinate transcription among rDNA repeat units [6, 7, 24, 31–35].

Concerted copy number variation (cCNV) refers to the correlation in copy number of 5S and 45S rDNA [36]. This co-variation in copy number across genotypes with variable rDNA array size is observed in human lymphoblastoid cells (LCLs) and occurs despite 5S and 45S rDNA residence on different chromosomes and lack of sequence homology between 5S and 45S rDNA subunits. Therefore, physical linkage between loci cannot explain the co-variation. On the other hand, spatial co-localization of the arrays as well as cellular processes of recombination such as those of micro-homology mediated end joining could conceivably contribute to the emergence of cCNV. Our results, however, confirmed a lack of direct 5S-45S contacts in Hi-C, an observation that is in agreement with a previous study [24]. This included a lack of 45S rDNA contacts with genes that are adjacent to the 5S rDNA array. The gene RHOU, for instance, is located next to the 5S and emerged in the bottom 3% of the distribution of 45S rDNA contact density. This indicates that the 5S and 45S rDNA are not in close enough proximity or that large protein complexes prevent the formation of 5S-45S Hi-C reads. The findings support the hypothesis that physical interactions occurring between 5S and 45S rDNA arrays are more restricted than previously anticipated. On the other hand, the denser maps presented here indicate that the 5S and 45S arrays share overlapping contact maps and many regions of the genome display a high density of contacts with both rDNA arrays. For instance, the density of 5S and 45S contacts is strongly correlated across genes and 1MB segments in both LCL and K562 cells. Whether or not this overlapping contact map is relevant for cCNV remains to be determined, but the evidence suggests that the two arrays are not completely independent. Coordination between them is likely to be relevant, with costs and benefits to 5S array proximity with the 45S arrays [24]. All in all, the association between contact density for the 5S and 45S arrays suggest that cCNV might be facilitated by structural proximity. Similarly, rDNA mediated structural changes in the nucleus might partially explain the regulatory consequences of naturally occurring variation in rDNA copy number [21].

From an evolutionary perspective, the co-existence of two clusters of rDNA loci (5S and 45S) might incur costs and benefits compared to rDNA residency on a single location. In some plants and yeasts, the 5S and 45S/35S rDNA subunits are spatially adjacent in the genome [7, 37–41], whereas in Drosophila and mammals, the 5S and 45S arrays reside on different chromosomes. However, the correlated contact maps for the 45S and 5S rDNA arrays suggest that they preferentially anchor at overlapping domains. This might narrow their spatial distances, and could explain why the 5S and 45S arrays can display apparent proximity to one another in a fraction of the cells as observed in cytological preparations. However, the lack of direct Hi-C 5S-45S contacts might suggest a model of competitive exclusion for similar anchoring sites, and predicts that a segment is in close proximity to either the 5S or 45S rDNA at each time. In cases of cytological proximity between the 5S and 45S arrays, large protein complexes might be present and prevent the emergence of direct 5S-45S inter-chromosomal contacts in the scale captured by Hi-C technology. Furthermore, the enrichment of rDNA contacts with ribosomal protein coding genes is surprising and might help explain the association between rDNA CN and the expression of these genes [21]. It suggests a structural component to the regulatory role of the rDNA and raises the possibility that the arrays might exert direct modulation of some genes via changes in nuclear organization. The data suggest that models that exclusively consider proximity to the rDNA arrays/nucleolus as a repressive modifier of gene expression might be overly simplistic. Rather, the distal and proximal association of genes with the rDNA arrays appears functionally motivated, as in the case of developmental genes or ribosomal genes. For instance, ribosomal gene proximity to the rDNA arrays could help facilitate coordinated Pol I, Pol II and Pol III responses. Collectively, these structural rDNA-mediated associations might have partially evolved to mitigate the fitness costs of dosage imbalances among highly expressed RNA and protein components of the translational machinery.

## Methods

### The 5S and 45S arrays

The human 5S rDNA along with flanking regions (chr1: 228,765,135–228,767,255) and the human 45S rDNA (GenBank reference number U13369.1, with modifications) were obtained as recently described [21, 36, 42, 43]. The 45S reference comprises the 18S, 5.8S and 28S rRNA encoding segments, external transcribed sequences (ETS) and internal transcribed segments 1 and 2 (ITS1 and ITS2), as well as a ~32 Kb non-coding intergenic spacer (IGS). Both 5S and 45S segments contain repetitive elements, such as Alu and Line1; all analysis carried out in this study used 5S and 45S sequences masked for these repeats.

### Hi-C data sets

Raw Hi-C reads for LCLs and erythroleukemia K562 (K562) cells were downloaded from the Gene Expression Omnibus (GEO) repository with accession number GSE63525 [44]. Biological replicates with more than 1 technical replicate were included for a total of 6,017,877,658 reads in LCL and 1,366,228,845 reads in K562. In addition, raw Hi-C reads for five cell types were obtained from GEO data with SRA Study number SRP033089 [45]. The five cell types comprised the H1 embryonic stem (ES) cells and four differentiated cell-types derived from H1 [Mesendoderm (ME) cells, Mesenchymal stem (MS) cells, Neuronal Progenitor (NP) cells, trophoblast-like (TB) cells] [45, 46]. The number of reads studied and recovery rates for 5S and 45S informative reads was summarized in Table 1.

### Data preparation and mapping

All data were downloaded in SRA format and converted into FASTQ files by the NCBI SRA Toolkit’s command (fastq-dump). FASTQ files were quality and adapter trimmed with Trim Galore. The trimming criteria required minimal quality score (> 20) and length (>50 bp). Next, we identified Hi-C reads that mapped to the 5S rDNA array or the 45S rDNA array. In this step, both forward and reverse reads were mapped independently to the 5S rDNA and 45S rDNA using Bowtie2 [47]. We used unpaired mapping with ‘very-sensitive’ mode (combinations of parameters: -D 20 -R 3 -N 0 -L 20 -i S, 1, 0.50). The mapping results were sorted and converted into binary format using SAMtools [48] and bed format using BEDTools [49]. We then extracted reads that mapped to the rDNA array and mapped the opposite end to repeat libraries. Reads for which one end mapped to repeats library were excluded. Finally, in order to identify potential confounders due to rDNA pseudogenes, both rDNA references were blasted against the human genome separately. Putative pseudogenes were identified as significant hits (*E*-value <1 × 10^−4^) using BLASTN [50, 51]. A segment of 1 MB was excluded from the analysis if an rDNA blast hit is identified within it. Similarly, a gene was removed from analysis if an rDNA blast hit is identified within its boundaries.

### Detecting genome-wide physical interaction with rDNA loci at 1MB resolution

To identify spatial variation in genomic contact density along the chromosomes we segmented the human genome GRCh37/hg19 assembly into 3,173 bins of 1MB using BEDTools [49]. Bins with rDNA pseudogenes were excluded. Contact densities were summarized for each bin for each of 5 cell types and 2 cell lines. We calculated the number of Contacts Per Million reads (CPM) to normalize the data and control for different number of reads in each of the seven conditions. This placed all the data in a comparable scale, to enable visualization of contact density along the human genome using heat maps in the 'gplots' R package [52].

### Analyses of rDNA-gene contacts

The term of “rDNA-gene contact” refers to reads with one end mapped to rDNA arrays and the other end mapped between the first and the last exon of an annotated gene in the human genome. We extracted coordinates of these reads using BEDTools [49] and the Gene Transfer Format (GTF) file: Homo_sapiens.GRCh37.75.gtf from the Ensembl database. GC content and length were also computed for each gene. To normalize contact densities in genes of different length, we computed the number of contacts per gene length in nucleotides (Contacts reads per gene per nucleotide, CPGN). The web based tool DAVID v6.8 [53] was used to investigate gene ontology enrichments for the top 5% of genes with greater CPGN for 5S rDNA or 45S rDNA genes. This corresponds to 494 out of 9864 genes for 5S-gene contacts, and 480 out of 9595 genes for 45S-gene contacts. The “one proportion” test [54] was also applied to address whether the number of mapped reads per base pair within a gene is significantly different from the genome wide average.

### Statistical analysis of differential contact density between LCL and K562 cells

We modeled differential contact density per 1MB and per gene using the edgeR package and statistical approaches adapted from RNA-seq analysis [55, 56]. Raw counts for physical contacts with rDNA loci within each bin along the human genome are modeled using generalized linear models (likelihood ratio tests) implemented in the edgeR package [55, 56]. These approaches were recently been used to detect differential interaction density (DIs) in Hi-C data [19, 57, 58]. The models identified statistically significant differences among cell lines/types in rDNA contacts density per MB and within genes. The Benjamini-Hochberg method was used for multiple testing correction [59], and statistical significance was denoted by FDR < 0.05. We applied the method to ascertain significant differences between LCL and K562 data from a single publication. For statistical comparison, we focused specifically on 11 biological replicates for LCL (collected with the Mbol enzyme) contrasted with two biological replicates for K562 (collected with the Mbol enzyme) and two biological replicates for LCL (collected with the DpnII enzyme). Each biological replicate consists of multiple technical replicates. We also evaluated variation among the five ES derived cell types, each with two biological replicates.

### Functional annotation of rDNA contacts

We cross-referenced the rDNA contact map with several sources of functional annotation. First, Hi-C studies proposed the partition of the genome into A and B compartments that are widely interpreted as open and closed chromatin, respectively [60]. A/B coordinates were downloaded for LCL cells and 12 cancer types [60]. Second, coordinates of 15 functional regions identified in hESC using ChromHMM [61] were downloaded. Third, information on replication timing along the genome was downloaded from the Replication Domain Database (www.replicationdomain.org). Finally, CTCF binding coordinates were obtained from the CTCFBSDB database [62]. We extracted the coordinates for all the segments in each annotation and addressed its density of rDNA contacts. BEDTools was used to assess the number of mapped reads that overlapped with each annotated segment for each dataset. The percentage of mapped reads was calculated by dividing the number of reads mapped to the segment by the total number of mapped reads. The genome wide average read per base pair was used to compute the expected number of reads in the functional segment. Statistical significance was assessed with Chi-square tests. In addition, we applied the “one proportion” statistical test [54] to address whether the numbers of mapped reads per base pair within a functional segment (e.g., CTCF binding) is significantly different from the genome-wide average per nucleotide contact rate.

## Supporting information

S1 Fig Comparison of results for positions of 45S rDNA contacts with Hi-C and nucleolus-associated chromatin domains (NADs). (A) part of chromosome 2q (chr2:90,052,599–169,971,888). (B) part of chromosome 15 q arm (chr15:19,990,398–63,840,300). Red boxes represent NADs [17], blue bars represent satellite repeats, orange box represent centromeres and blue horizontal bars represent a part of chromosome 2 or 15. Sites of 45S Hi-C contacts recovered with LCL and K562 are shown.
(TIF)

S2 Fig (A) Plots of expected vs. observed number of hits per chromosome for LCL cell types. (B) Plots of expected vs. observed number of hits per chromosome for K562 cell types. (C) Plots of expected vs. observed number of hits per chromosome for ESC related cell types.
(TIF)

S3 Fig Reproducibility of 45S rDNA contacts in technical replicates (1 to 9) of the LCL set. Each dot represents the number of contacts identified in each 1MB segment. Red lines in the lower panels are loess smoothers. Upper panels show the spearman rank correlation between datasets. All correlations are statistically significant (P < 0.001).
(TIF)

S4 Fig Reproducibility of 45S rDNA contacts in technical replicates (10 to 18) of the LCL set. Each dot represents the number of contacts identified in each 1MB segment. Red lines in the lower panels are loess smoothers. Upper panels show the spearman rank correlation between datasets. All correlations are statistically significant (P < 0.001).
(TIF)

S5 Fig Reproducibility of 45S rDNA contacts in biological (set 32 and 33) and technical replicates of the LCL set. Each dot represents the number of contacts identified in each 1MB segment. Red lines in the lower panels are loess smoothers. Upper panels show the spearman rank correlation between datasets. All correlations are statistically significant (P < 0.001).
(TIF)

S6 Fig Reproducibility of 45S rDNA contacts across biological replicates with the same enzyme (replicate 32 and 33 with DpnII) and with different enzymes (replicate 1 with Mbol). Data for the LCL set, with all technical replicates combined in each biological replicate. Each dot represents the number of contacts identified in each 1MB segment. Red lines in the lower panels are loess smoothers. Upper panels show the spearman rank correlation between datasets. All correlations are statistically significant (P < 0.001).
(TIF)

S7 Fig Reproducibility of 45S rDNA contacts across biological replicates. Data for the LCL set, with all technical replicates combined in each biological replicate. Each dot represents the number of contacts identified in each 1MB segment. Red lines in the lower panels are loess smoothers. Upper panels show the spearman rank correlation between datasets. All correlations are statistically significant (P < 0.001).
(TIF)

S8 Fig Reproducibility of 45S rDNA contacts across biological replicates. Data for K562, with all technical replicates combined in each biological replicate. Each dot represents the number of contacts identified in each 1MB segment. Red lines in the lower panels are loess smoothers. Upper panels show the spearman rank correlation between datasets. All correlations are statistically significant (P < 0.001).
(TIF)

S9 Fig Correlation of 45S rDNA contacts across two cell lines (LCL and K562) and five ESC derived cell types. All biological replicates combined. Each dot represents the number of contacts identified in each 1MB segment. Red lines in the lower panels are loess smoothers. Upper panels show the spearman rank correlation between datasets. All correlations are statistically significant (P < 0.001).
(TIF)

S10 Fig Ascertaining significant 45S rDNA contact density variation across genes. (A) Volcano plot for the contrast between biological replicates of LCL collected with different enzymes (Mbol and DpnII). (B) Volcano plot for the contrast between LCL vs K562 replicates collected with the same enzyme (Mbol). Green dots represent genes with > 2-fold change. Red dots represent segments with difference larger than 2-fold change (vertical line) and P-value < 3 × 10^−4^. (horizontal line).
(TIF)

S11 Fig Closed, repressed, late replicating chromatin segments are preferentially associated with the rDNA array. (A and B) Number of observed (black) and expected (white) rDNA contacts with each functional annotation for two sets of biological replicates in LCL. Expected numbers are calculated with the genome-wide per nucleotide contact rate. Shown is data for three selected annotations from 15-label genomic segmentation of hESC using ChromHMM [61].
(TIF)

S1 Table Summary table of Hi-C reads mapping to the 5S rDNA repeat unit (2121bp) in each dataset. Shown are the number of reads for which both ends map to the 5S repeat unit and the number of reads for which one end maps to the 5S rDNA repeat unit and the other maps to the rest of the genome [whole genome (WG)]. Both the 5S rDNA unit and the whole genome were masked for repetitive elements.
(TIF)

S2 Table Summary table of Hi-C reads mapping to the 45S rDNA repeat unit (45,337 bp) in each dataset. Shown are the number of reads for which both ends map to the 45S rDNA repeat unit and the number of reads for which one end maps to the 45S rDNA repeat unit and the other maps to the rest of the genome [whole genome (WG)]. Both the 45S rDNA unit and the whole genome were masked for repetitive elements.
(TIF)

S3 Table Number of 1MB segments displaying differential density of interactions with the 45S rDNA array in LCL and K562. Statistically significant segments ascertained with the EdgeR package. The number of segments with significant differences is shown for each chromosome.
(TIF)

S4 Table Number of 1MB segments displaying differential density of interactions with the 45S rDNA array among five ESC-related cell types. Statistically significant segments ascertained with the EdgeR package. The number of segments with significant differences is shown for each chromosome.
(TIF)

S5 Table Average number of contacts per nucleotide (CPN) obtained with three subsets of contacts. CPN obtained from (i) all rDNA-genome contacts (without controlling for pseudogenes), (ii) rDNA-genome contacts retrieved from 1MB bins without rDNA pseudogenes, and (iii) rDNA-gene contacts retrieved from genes without rDNA pseudogenes in them. Genome-wide contacts were divided by the genome size or the total size of the bins without pseudogenes. rDNA-gene contacts were divided by the total length of the genome with genic sequences (protein-coding genes only). Shown are estimates for both 5S and 45S rDNA arrays.
(TIF)

S6 Table Summary table of gene ontology enrichments for the top 5% of genes contacted with the 5S rDNA in the K562 and LCL cell types. Selected categories are shown.
(TIF)

S7 Table Summary table for gene ontology enrichment for the top 5% of genes contacted with the 45S rDNA in LCL. Raw and adjusted P-values are listed. Adjusted P-values were obtained after Benjamini-Hochberg correction. All categories with raw P-value < 0.05 are shown.
(TIF)

S8 Table Summary table for gene ontology enrichment for the top 5% of genes contacted with the 45S rDNA in K562. Raw and adjusted P-values are listed. Adjusted P-values were obtained after Benjamini-Hochberg correction. All categories with raw P-value < 0.05 are shown.
(TIF)

S9 Table Summary table for gene ontology enrichment for the top 5% of genes contacted with the 45S rDNA in the ESC set. Raw and adjusted P-values are listed. Adjusted P-values were obtained after Benjamini-Hochberg correction. All categories with raw P-value < 0.05 are shown.
(TIF)

## References

[1] Warner JR. The economics of ribosome biosynthesis in yeast. Trends Biochem Sci. 1999;24(11):437–40. Epub 1999/11/05. . 10542411 10.1016/s0968-0004(99)01460-7

[2] Grummt I. Life on a planet of its own: regulation of RNA polymerase I transcription in the nucleolus. Genes Dev. 2003;17(14):1691–702. Epub 2003/07/17. doi: 10.1101/gad.1098503R . 12865296 10.1101/gad.1098503R

[3] Pederson T. The nucleolus. Cold Spring Harb Perspect Biol. 2011;3(3). Epub 2010/11/26. doi: 10.1101/cshperspect.a000638 ; PubMed Central PMCID: PMC3039934. 21106648 10.1101/cshperspect.a000638 PMC3039934

[4] Woolford JL Jr., Baserga SJ. Ribosome biogenesis in the yeast Saccharomyces cerevisiae. Genetics. 2013;195(3):643–81. Epub 2013/11/06. doi: 10.1534/genetics.113.153197 ; PubMed Central PMCID: PMC3813855. 24190922 10.1534/genetics.113.153197 PMC3813855

[5] Henderson A, Warburton D, Atwood K. Location of ribosomal DNA in the human chromosome complement. Proceedings of the National Academy of Sciences. 1972;69(11):3394–8. 10.1073/pnas.69.11.3394 PMC389778 4508329

[6] Henderson A, Warburton D, Atwood K. Ribosomal DNA connectives between human acrocentric chromosomes. Proceedings of the National Academy of Sciences. 1972; 69(11):3394–3398. 10.1073/pnas.69.11.3394 PMC389778 4508329

[7] Wicke S, Costa A, Muñoz J, Quandt D. Restless 5S: The re-arrangement (s) and evolution of the nuclear ribosomal DNA in land plants. Molecular phylogenetics and evolution. 2011;61(2):321–32. doi: 10.1016/j.ympev.2011.06.023 21757016 10.1016/j.ympev.2011.06.023

[8] Moss T, Langlois F, Gagnon-Kugler T, Stefanovsky V. A housekeeper with power of attorney: the rRNA genes in ribosome biogenesis. Cell Mol Life Sci. 2007;64(1):29–49. Epub 2006/12/16. doi: 10.1007/s00018-006-6278-1 . 17171232 10.1007/s00018-006-6278-1 PMC11136161

[9] Fedoriw AM, Starmer J, Yee D, Magnuson T. Nucleolar association and transcriptional inhibition through 5S rDNA in mammals. PLoS Genet. 2012;8(1):e1002468 Epub 2012/01/26. doi: 10.1371/journal.pgen.1002468 ; PubMed Central PMCID: PMC3261910. 22275877 10.1371/journal.pgen.1002468 PMC3261910

[10] Smith CL, Matheson TD, Trombly DJ, Sun X, Campeau E, Han X, et al A separable domain of the p150 subunit of human chromatin assembly factor-1 promotes protein and chromosome associations with nucleoli. Mol Biol Cell. 2014;25(18):2866–81. Epub 2014/07/25. doi: 10.1091/mbc.E14-05-1029 ; PubMed Central PMCID: PMC4161520. 25057015 10.1091/mbc.E14-05-1029 PMC4161520

[11] Kaplan FS, Murray J, Sylvester JE, Gonzalez IL, O'Connor JP, Doering JL, et al The topographic organization of repetitive DNA in the human nucleolus. Genomics. 1993;15(1):123–32. Epub 1993/01/01. doi: 10.1006/geno.1993.1020 . 8432523 10.1006/geno.1993.1020

[12] Dekker J, Rippe K, Dekker M, Kleckner N. Capturing chromosome conformation. science. 2002;295(5558):1306–11. doi: 10.1126/science.1067799 11847345 10.1126/science.1067799

[13] van Berkum NL, Lieberman-Aiden E, Williams L, Imakaev M, Gnirke A, Mirny LA, et al Hi-C: a method to study the three-dimensional architecture of genomes. J Vis Exp. 2010;(39). Epub 2010/05/13. doi: 10.3791/1869 ; PubMed Central PMCID: PMC3149993. 20461051 10.3791/1869 PMC3149993

[14] Lieberman-Aiden E, van Berkum NL, Williams L, Imakaev M, Ragoczy T, Telling A, et al Comprehensive mapping of long-range interactions reveals folding principles of the human genome. Science. 2009;326(5950):289–93. Epub 2009/10/10. doi: 10.1126/science.1181369 ; PubMed Central PMCID: PMC2858594. 19815776 10.1126/science.1181369 PMC2858594

[15] Nora EP, Lajoie BR, Schulz EG, Giorgetti L, Okamoto I, Servant N, et al Spatial partitioning of the regulatory landscape of the X-inactivation centre. Nature. 2012;485(7398):381–5. doi: 10.1038/nature11049 22495304 10.1038/nature11049 PMC3555144

[16] Dixon JR, Selvaraj S, Yue F, Kim A, Li Y, Shen Y, et al Topological domains in mammalian genomes identified by analysis of chromatin interactions. Nature. 2012;485(7398):376–80. doi: 10.1038/nature11082 22495300 10.1038/nature11082 PMC3356448

[17] Nemeth A, Conesa A, Santoyo-Lopez J, Medina I, Montaner D, Peterfia B, et al Initial genomics of the human nucleolus. PLoS Genet. 2010;6(3):e1000889 Epub 2010/04/03. doi: 10.1371/journal.pgen.1000889 ; PubMed Central PMCID: PMC2845662. 20361057 10.1371/journal.pgen.1000889 PMC2845662

[18] Nemeth A, Langst G. Genome organization in and around the nucleolus. Trends Genet. 2011;27(4):149–56. Epub 2011/02/08. doi: 10.1016/j.tig.2011.01.002 . 21295884 10.1016/j.tig.2011.01.002

[19] Matheson TD, Kaufman PD. Grabbing the genome by the NADs. Chromosoma. 2016;125(3):361–71. Epub 2015/07/16. doi: 10.1007/s00412-015-0527-8 ; PubMed Central PMCID: PMC4714962. 26174338 10.1007/s00412-015-0527-8 PMC4714962

[20] van de Nobelen S, Rosa-Garrido M, Leers J, Heath H, Soochit W, Joosen L, et al CTCF regulates the local epigenetic state of ribosomal DNA repeats. Epigenetics & chromatin. 2010;3(1):19. 21059229 10.1186/1756-8935-3-19 PMC2993708

[21] Gibbons JG, Branco AT, Yu S, Lemos B. Ribosomal DNA copy number is coupled with gene expression variation and mitochondrial abundance in humans. Nature communications. 2014;5:4850 doi: 10.1038/ncomms5850 . 25209200 10.1038/ncomms5850

[22] Bell SP, Learned RM, Jantzen H-M, Tjian R. Functional cooperativity between transcription factors UBF1 and SL1 mediates human ribosomal RNA synthesis. Science. 1988;241(4870):1192–7. 3413483 10.1126/science.3413483

[23] Li P, Gao S, Wang L, Yu F, Li J, Wang C, et al ABH2 couples regulation of ribosomal DNA transcription with DNA alkylation repair. Cell reports. 2013;4(4):817–29. doi: 10.1016/j.celrep.2013.07.027 23972994 10.1016/j.celrep.2013.07.027

[24] Yu S, Lemos B. A portrait of ribosomal DNA contacts with Hi-C reveals 5S and 45S rDNA anchoring points in the folded human genome. Genome biology and evolution. 2016;8(11):3545–58. doi: 10.1093/gbe/evw257 27797956 10.1093/gbe/evw257 PMC5203791

[25] Naumann S, Reutzel D, Speicher M, Decker HJ. Complete karyotype characterization of the K562 cell line by combined application of G-banding, multiplex-fluorescence in situ hybridization, fluorescence in situ hybridization, and comparative genomic hybridization. Leuk Res. 2001;25(4):313–22. . 11248328 10.1016/s0145-2126(00)00125-9

[26] O'Sullivan JM, Pai DA, Cridge AG, Engelke DR, Ganley AR. The nucleolus: a raft adrift in the nuclear sea or the keystone in nuclear structure? Biomol Concepts. 2013;4(3):277–86. doi: 10.1515/bmc-2012-0043 ; PubMed Central PMCID: PMCPMC5100006. 25436580 10.1515/bmc-2012-0043 PMC5100006

[27] Paredes S, Branco AT, Hartl DL, Maggert KA, Lemos B. Ribosomal DNA deletions modulate genome-wide gene expression: "rDNA-sensitive" genes and natural variation. PLoS Genet. 2011;7(4):e1001376 Epub 2011/05/03. doi: 10.1371/journal.pgen.1001376 ; PubMed Central PMCID: PMC3080856. 21533076 10.1371/journal.pgen.1001376 PMC3080856

[28] Magalhaes PJ, Andreu AL, Schon EA. Evidence for the presence of 5S rRNA in mammalian mitochondria. Mol Biol Cell. 1998;9(9):2375–82. Epub 1998/09/03. ; PubMed Central PMCID: PMC25503. 9725900 10.1091/mbc.9.9.2375 PMC25503

[29] Smirnov A, Entelis N, Martin RP, Tarassov I. Biological significance of 5S rRNA import into human mitochondria: role of ribosomal protein MRP-L18. Genes Dev. 2011;25(12):1289–305. Epub 2011/06/21. doi: 10.1101/gad.624711 ; PubMed Central PMCID: PMC3127430. 21685364 10.1101/gad.624711 PMC3127430

[30] Cahyani I, Cridge AG, Engelke DR, Ganley AR, O'Sullivan JM. A sequence-specific interaction between the Saccharomyces cerevisiae rRNA gene repeats and a locus encoding an RNA polymerase I subunit affects ribosomal DNA stability. Mol Cell Biol. 2015;35(3):544–54. Epub 2014/11/26. doi: 10.1128/MCB.01249-14 ; PubMed Central PMCID: PMC4285424. 25421713 10.1128/MCB.01249-14 PMC4285424

[31] Raška I, Shaw PJ, Cmarko D. New insights into nucleolar architecture and activity. International review of cytology. 2006;255:177–235. doi: 10.1016/S0074-7696(06)55004-1 17178467 10.1016/S0074-7696(06)55004-1

[32] Bouteille M, Kalifat SR, Delarue J. Ultrastructural variations of nuclear bodies in human diseases. J Ultrastruct Res. 1967;19(5):474–86. Epub 1967/08/30. . 4293463 10.1016/s0022-5320(67)80074-1

[33] Goessens G. Nucleolar structure. Int Rev Cytol. 1984;87:107–58. Epub 1984/01/01. . 6201455 10.1016/s0074-7696(08)62441-9

[34] Fischer D, Weisenberger D, Scheer U. Assigning functions to nucleolar structures. Chromosoma. 1991;101(3):133–40. Epub 1991/12/01. . 1790729 10.1007/BF00355363

[35] Scheer U, Thiry M, Goessens G. Structure, function and assembly of the nucleolus. Trends Cell Biol. 1993;3(7):236–41. Epub 1993/07/01. . 14731759 10.1016/0962-8924(93)90123-i

[36] Gibbons JG, Branco AT, Godinho SA, Yu S, Lemos B. Concerted copy number variation balances ribosomal DNA dosage in human and mouse genomes. Proceedings of the National Academy of Sciences. 2015; 112(8):2485–2490. doi: 10.1073/pnas.1416878112 . 25583482 10.1073/pnas.1416878112 PMC4345604

[37] Petes TD. Yeast ribosomal DNA genes are located on chromosome XII. Proceedings of the National Academy of Sciences. 1979;76(1):410–4. 10.1073/pnas.76.1.410 PMC382949 370829

[38] Sone T, Fujsawa M, Takanaka M, Nakagawa S, Yamaoka S, Sakaida M, et al Bryophyte 5S was inserted into 45S rDNA repeat units after the divergence from higher land plants. Plant Molecular Biolology. 1999;41:679–85. 10.1023/a:1006398419556 10645727

[39] Ganley AR, Kobayashi T. Highly efficient concerted evolution in the ribosomal DNA repeats: total rDNA repeat variation revealed by whole-genome shotgun sequence data. Genome Research. 2007;17(2):184–91. doi: 10.1101/gr.5457707 17200233 10.1101/gr.5457707 PMC1781350

[40] Liu Y, Forrest LL, Bainard JD, Budke JM, Goffinet B. Organellar genome, nuclear ribosomal DNA repeat unit, and microsatellites isolated from a small-scale of 454 GS FLX sequencing on two mosses. Mol Phylogenet Evol. 2013;66(3):1089–94. Epub 2012/12/25. doi: 10.1016/j.ympev.2012.12.006 . 23261712 10.1016/j.ympev.2012.12.006

[41] Garcia S, Galvez F, Gras A, Kovarik A, Garnatje T. Plant rDNA database: update and new features. Database (Oxford). 2014;2014 Epub 2014/07/02. doi: 10.1093/database/bau063 ; PubMed Central PMCID: PMC4075780. 24980131 10.1093/database/bau063 PMC4075780

[42] Gonzalez IL, Sylvester JE. Complete sequence of the 43-kb human ribosomal DNA repeat: analysis of the intergenic spacer. Genomics. 1995;27(2):320–8. doi: 10.1006/geno.1995.1049 7557999 10.1006/geno.1995.1049

[43] Zentner GE, Saiakhova A, Manaenkov P, Adams MD, Scacheri PC. Integrative genomic analysis of human ribosomal DNA. Nucleic acids research. 2011:gkq1326. 10.1093/nar/gkq1326 PMC3130253 21355038

[44] Rao SS, Huntley MH, Durand NC, Stamenova EK, Bochkov ID, Robinson JT, et al A 3D map of the human genome at kilobase resolution reveals principles of chromatin looping. Cell. 2014;159(7):1665–80. Epub 2014/12/17. doi: 10.1016/j.cell.2014.11.021 . 25497547 10.1016/j.cell.2014.11.021 PMC5635824

[45] Dixon JR, Jung I, Selvaraj S, Shen Y, Antosiewicz-Bourget JE, Lee AY, et al Chromatin architecture reorganization during stem cell differentiation. Nature. 2015;518(7539):331–6. doi: 10.1038/nature14222 25693564 10.1038/nature14222 PMC4515363

[46] Xie W, Schultz MD, Lister R, Hou Z, Rajagopal N, Ray P, et al Epigenomic analysis of multilineage differentiation of human embryonic stem cells. Cell. 2013;153(5):1134–48. doi: 10.1016/j.cell.2013.04.022 23664764 10.1016/j.cell.2013.04.022 PMC3786220

[47] Langmead B, Salzberg SL. Fast gapped-read alignment with Bowtie 2. Nature methods. 2012;9(4):357–9. doi: 10.1038/nmeth.1923 22388286 10.1038/nmeth.1923 PMC3322381

[48] Li H, Handsaker B, Wysoker A, Fennell T, Ruan J, Homer N, et al The sequence alignment/map format and SAMtools. Bioinformatics. 2009;25(16):2078–9. doi: 10.1093/bioinformatics/btp352 19505943 10.1093/bioinformatics/btp352 PMC2723002

[49] Quinlan AR, Hall IM. BEDTools: a flexible suite of utilities for comparing genomic features. Bioinformatics. 2010;26(6):841–2. doi: 10.1093/bioinformatics/btq033 20110278 10.1093/bioinformatics/btq033 PMC2832824

[50] Altschul SF, Gish W, Miller W, Myers EW, Lipman DJ. Basic local alignment search tool. Journal of molecular biology. 1990;215(3):403–10. doi: 10.1016/S0022-2836(05)80360-2 2231712 10.1016/S0022-2836(05)80360-2

[51] Altschul SF, Madden TL, Schäffer AA, Zhang J, Zhang Z, Miller W, et al Gapped BLAST and PSI-BLAST: a new generation of protein database search programs. Nucleic acids research. 1997;25(17):3389–402. 9254694 10.1093/nar/25.17.3389 PMC146917

[52] Warnes GR, Bolker B, Bonebakker L, Gentleman R, Huber W, Liaw A, et al gplots: Various R programming tools for plotting data. R package version. 2009;2(4):1.

[53] Huang DW, Sherman BT, Lempicki RA. Systematic and integrative analysis of large gene lists using DAVID bioinformatics resources. Nature protocols. 2009;4(1):44–57. doi: 10.1038/nprot.2008.211 19131956 10.1038/nprot.2008.211

[54] Altman DG. Practical statistics for medical research: CRC press; 1990.

[55] McCarthy DJ, Chen Y, Smyth GK. Differential expression analysis of multifactor RNA-Seq experiments with respect to biological variation. Nucleic acids research. 2012;40(10):4288–97. doi: 10.1093/nar/gks042 22287627 10.1093/nar/gks042 PMC3378882

[56] Robinson MD, McCarthy DJ, Smyth GK. edgeR: a Bioconductor package for differential expression analysis of digital gene expression data. Bioinformatics. 2010;26(1):139–40. doi: 10.1093/bioinformatics/btp616 19910308 10.1093/bioinformatics/btp616 PMC2796818

[57] Paulsen J, Sandve GK, Gundersen S, Lien TG, Trengereid K, Hovig E. HiBrowse: multi-purpose statistical analysis of genome-wide chromatin 3D organization. Bioinformatics. 2014;30(11):1620–2. doi: 10.1093/bioinformatics/btu082 24511080 10.1093/bioinformatics/btu082 PMC4029040

[58] Lun AT, Smyth GK. diffHic: a Bioconductor package to detect differential genomic interactions in Hi-C data. BMC bioinformatics. 2015;16(1):258. 26283514 10.1186/s12859-015-0683-0 PMC4539688

[59] Benjamini Y, Hochberg Y. Controlling the false discovery rate: a practical and powerful approach to multiple testing. Journal of the royal statistical society Series B (Methodological). 1995:289–300.

[60] Fortin JP, Hansen KD. Reconstructing A/B compartments as revealed by Hi-C using long-range correlations in epigenetic data. Genome biology. 2015;16:180 Epub 2015/09/01. doi: 10.1186/s13059-015-0741-y ; PubMed Central PMCID: PMC4574526. 26316348 10.1186/s13059-015-0741-y PMC4574526

[61] Ernst J, Kellis M. ChromHMM: automating chromatin-state discovery and characterization. Nature methods. 2012;9(3):215–6. Epub 2012/03/01. doi: 10.1038/nmeth.1906 ; PubMed Central PMCID: PMC3577932. 22373907 10.1038/nmeth.1906 PMC3577932

[62] Ziebarth JD, Bhattacharya A, Cui Y. CTCFBSDB 2.0: a database for CTCF-binding sites and genome organization. Nucleic acids research. 2012;41(D1):D188–D94. 23193294 10.1093/nar/gks1165 PMC3531215

