# Supplementary figures and images for: The long-range interaction map of ribosomal DNA arrays
Source: PLoS Genet. 2018 Mar 23;14(3):e1007258. doi: 10.1371/journal.pgen.1007258 (PMC5865718; doi:10.1371/journal.pgen.1007258)

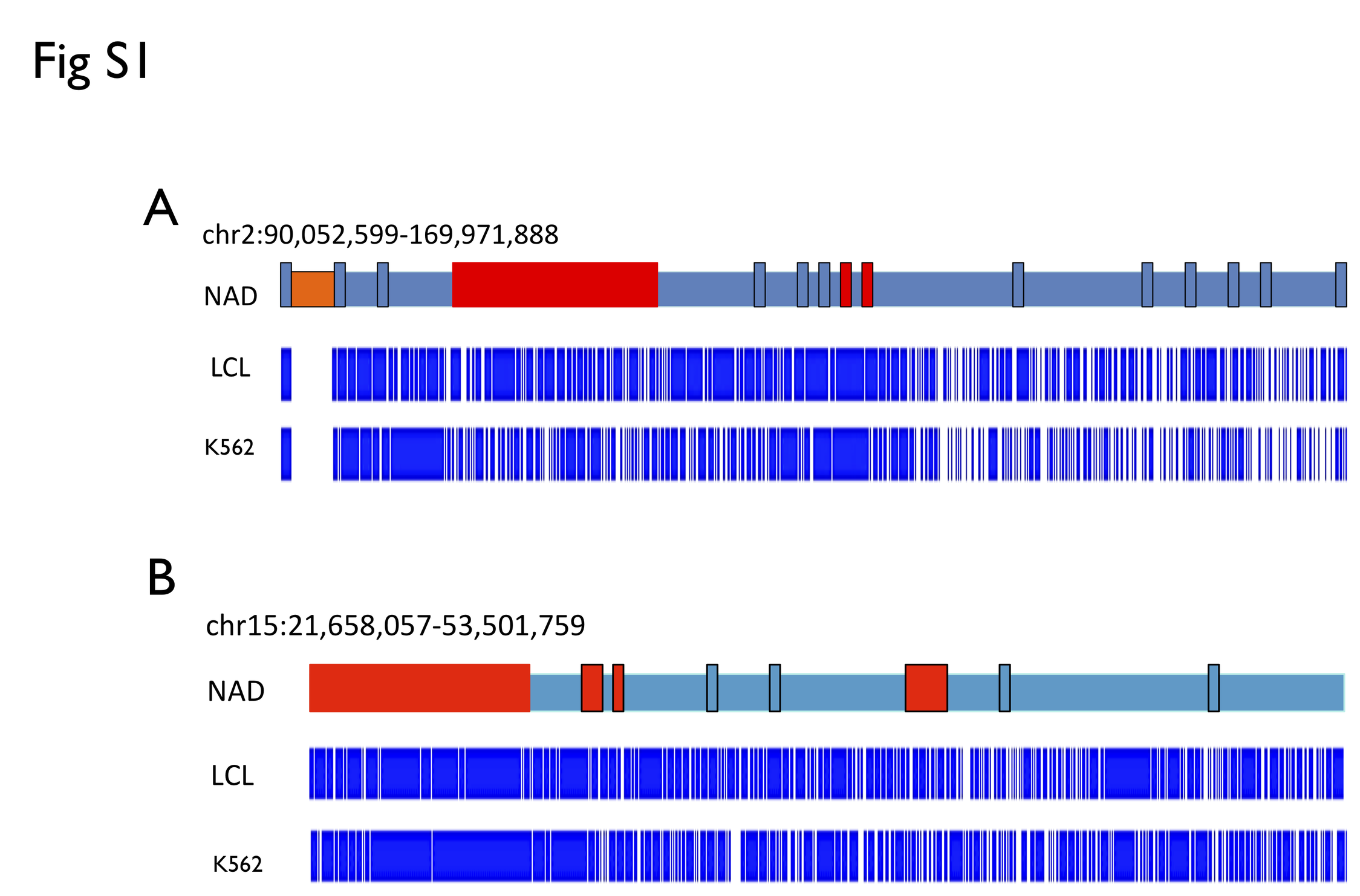

Supplement: S1 Fig — (A) part of chromosome 2q (chr2:90,052,599–169,971,888). (B) part of chromosome 15 q arm (chr15:19,990,398–63,840,300). Red boxes represent NADs [17], blue bars represent satellite repeats, orange box represent centromeres and blue horizontal bars represent a part of chromosome 2 or 15. Sites of 45S Hi-C contacts recovered with LCL and K562 are shown. (TIF) [file pgen.1007258.s001.tif]

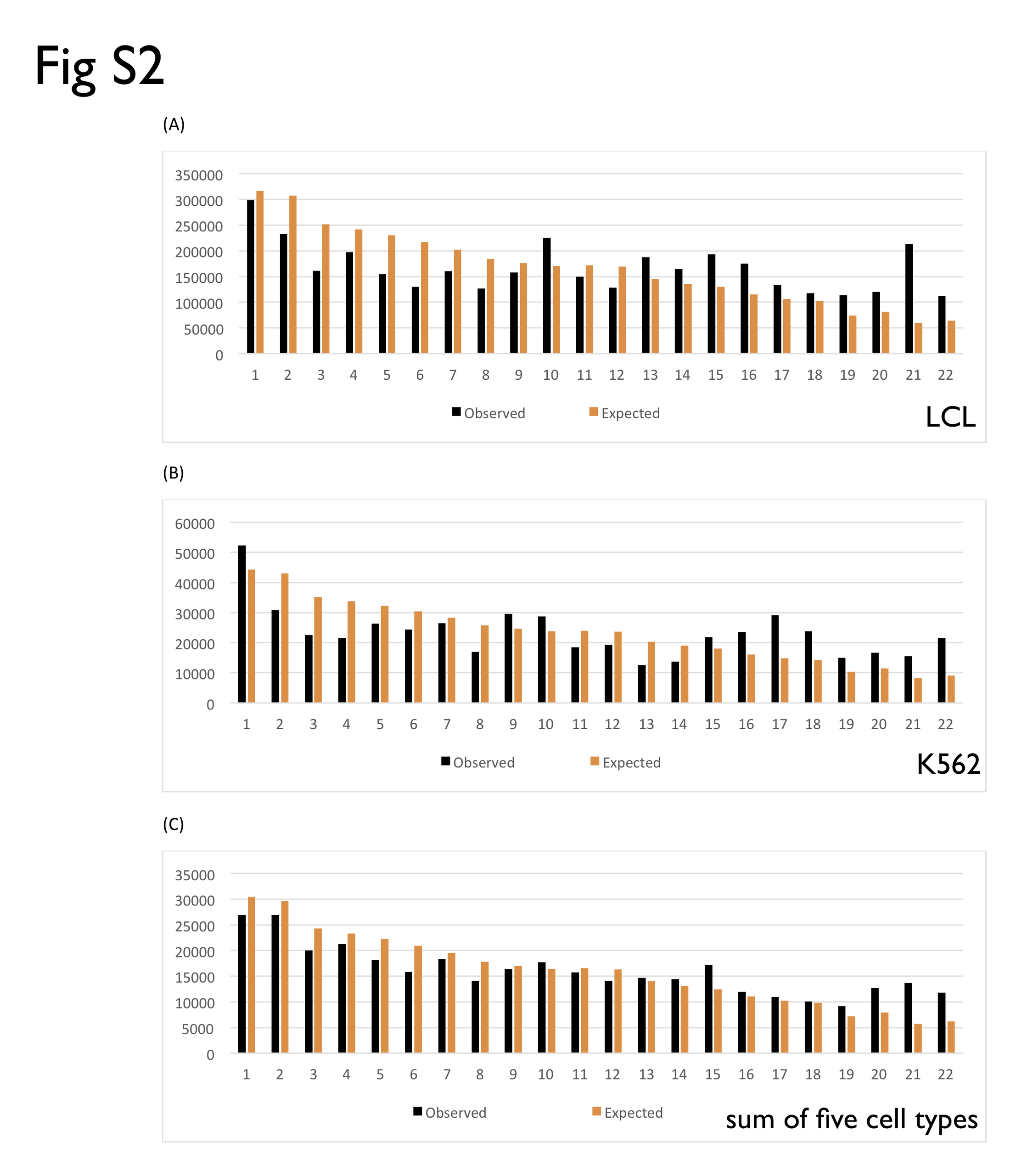

Supplement: S2 Fig — (A) Plots of expected vs. observed number of hits per chromosome for LCL cell types. (B) Plots of expected vs. observed number of hits per chromosome for K562 cell types. (C) Plots of expected vs. observed number of hits per chromosome for ESC related cell types. (TIF) [file pgen.1007258.s002.tif]

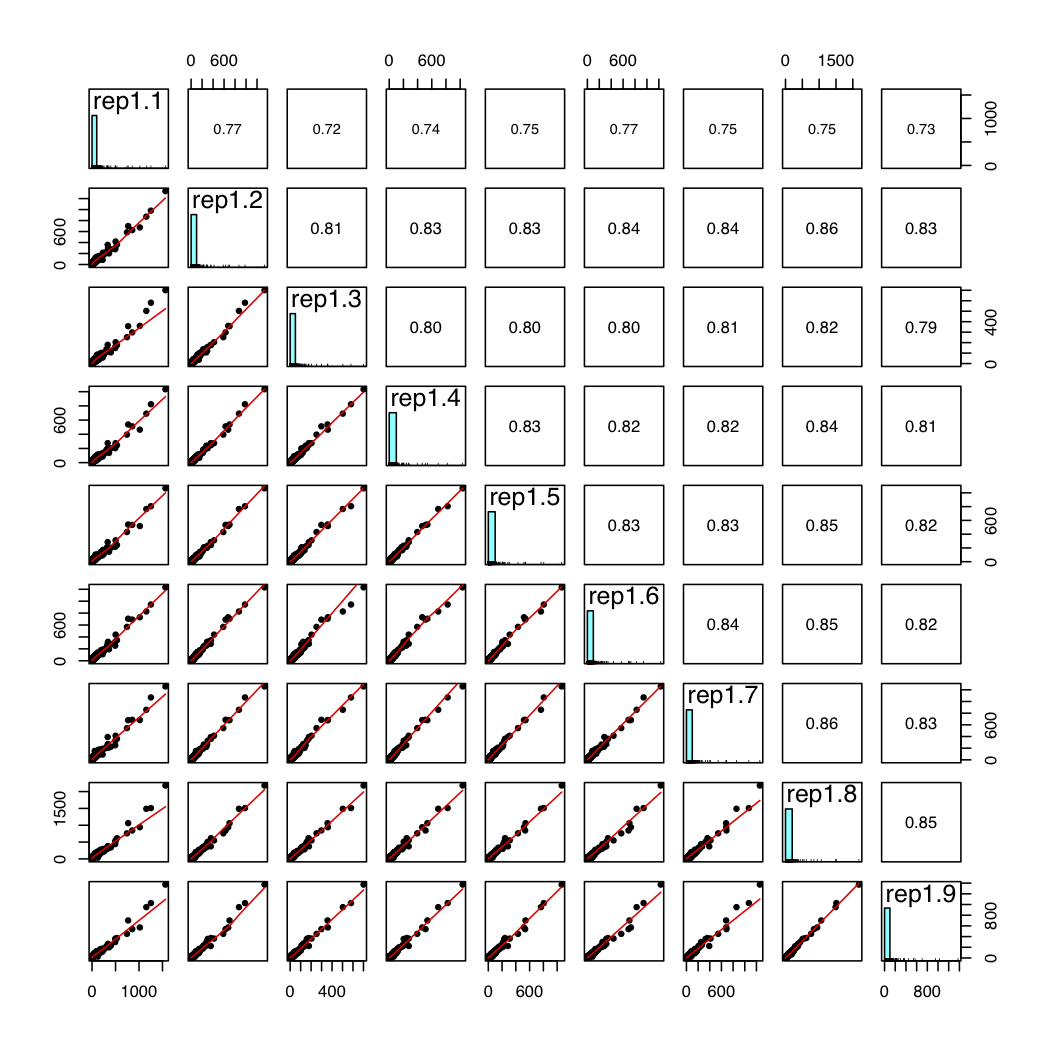

Supplement: S3 Fig — Each dot represents the number of contacts identified in each 1MB segment. Red lines in the lower panels are loess smoothers. Upper panels show the spearman rank correlation between datasets. All correlations are statistically significant (P < 0.001). (TIF) [file pgen.1007258.s003.tif]

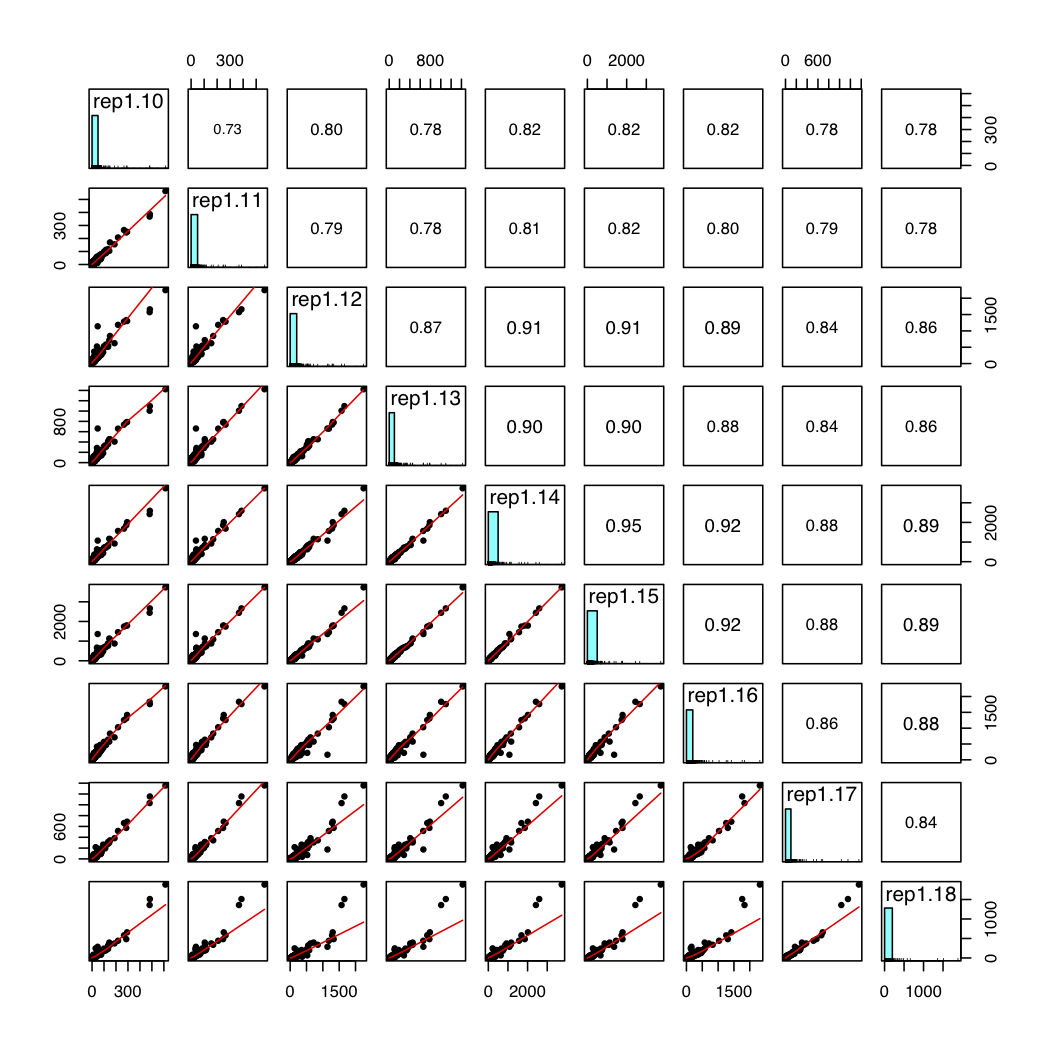

Supplement: S4 Fig — Each dot represents the number of contacts identified in each 1MB segment. Red lines in the lower panels are loess smoothers. Upper panels show the spearman rank correlation between datasets. All correlations are statistically significant (P < 0.001). (TIF) [file pgen.1007258.s004.tif]

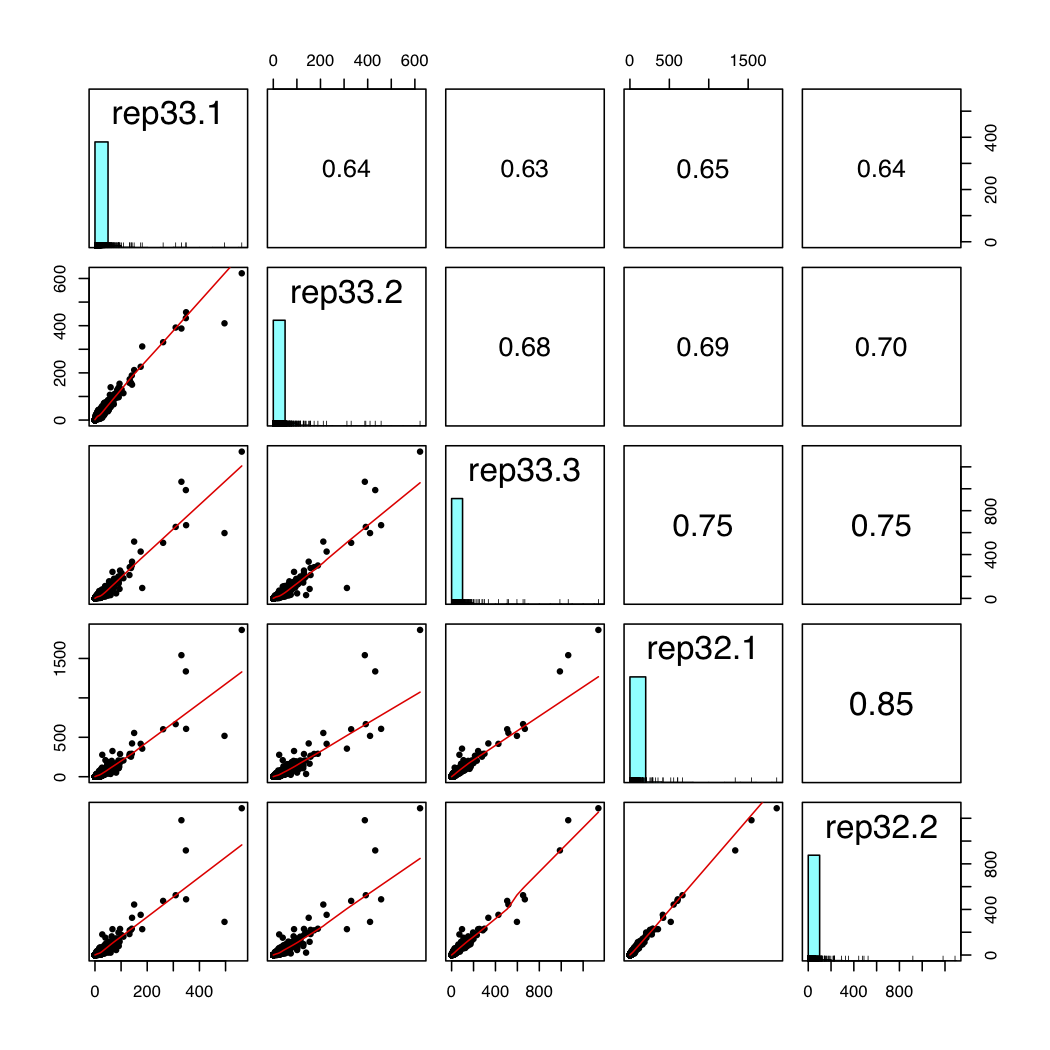

Supplement: S5 Fig — Each dot represents the number of contacts identified in each 1MB segment. Red lines in the lower panels are loess smoothers. Upper panels show the spearman rank correlation between datasets. All correlations are statistically significant (P < 0.001). (TIF) [file pgen.1007258.s005.tif]

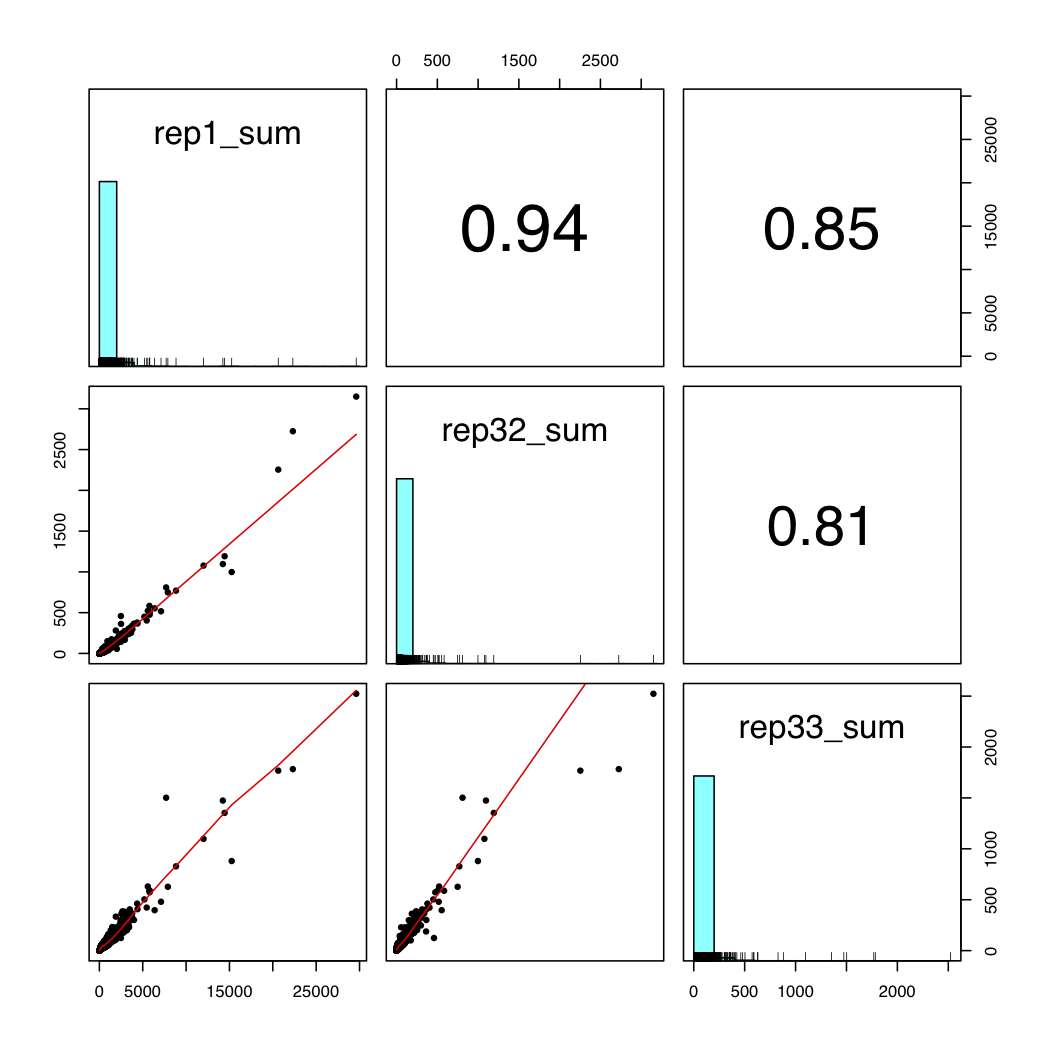

Supplement: S6 Fig — Data for the LCL set, with all technical replicates combined in each biological replicate. Each dot represents the number of contacts identified in each 1MB segment. Red lines in the lower panels are loess smoothers. Upper panels show the spearman rank correlation between datasets. All correlations are statistically significant (P < 0.001). (TIF) [file pgen.1007258.s006.tif]

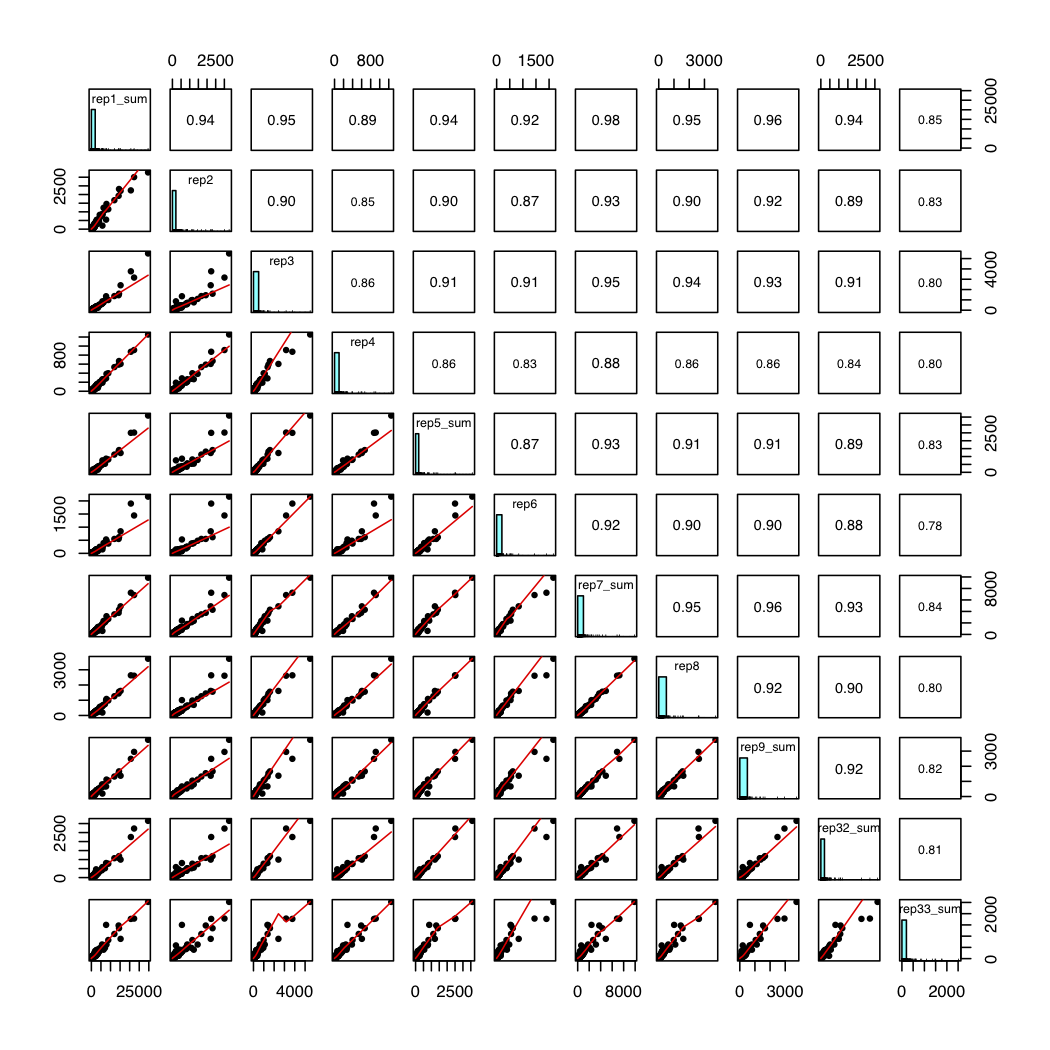

Supplement: S7 Fig — Data for the LCL set, with all technical replicates combined in each biological replicate. Each dot represents the number of contacts identified in each 1MB segment. Red lines in the lower panels are loess smoothers. Upper panels show the spearman rank correlation between datasets. All correlations are statistically significant (P < 0.001). (TIF) [file pgen.1007258.s007.tif]

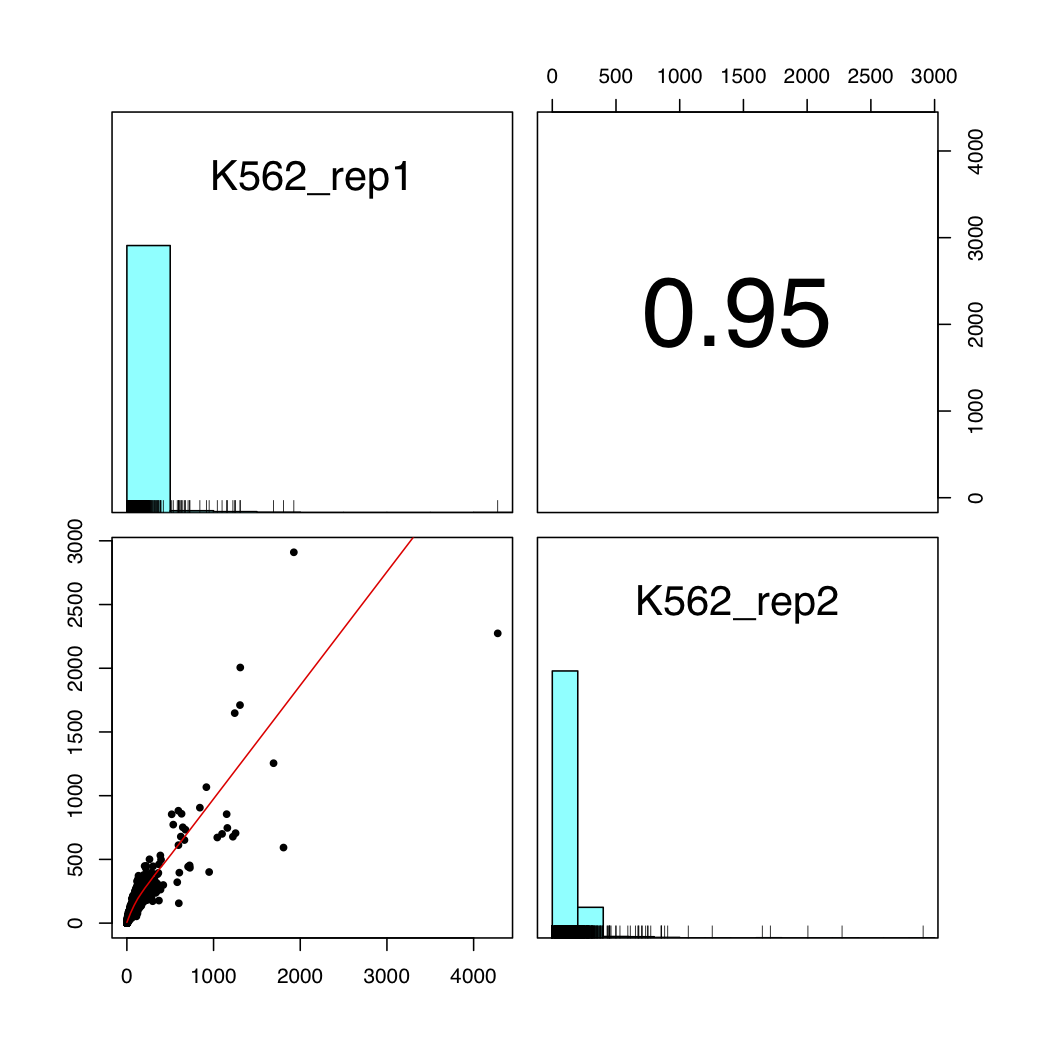

Supplement: S8 Fig — Data for K562, with all technical replicates combined in each biological replicate. Each dot represents the number of contacts identified in each 1MB segment. Red lines in the lower panels are loess smoothers. Upper panels show the spearman rank correlation between datasets. All correlations are statistically significant (P < 0.001). (TIF) [file pgen.1007258.s008.tif]

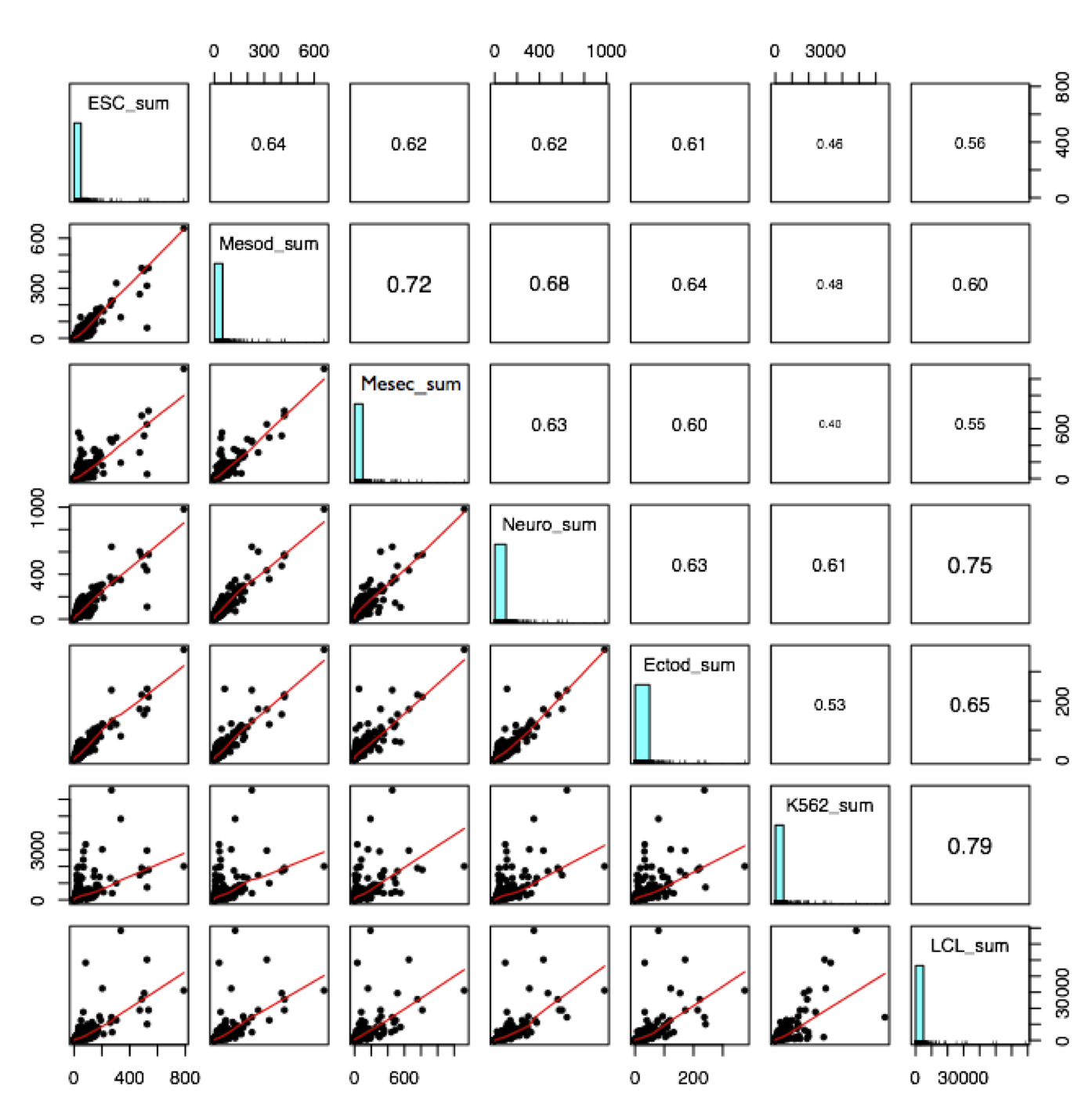

Supplement: S9 Fig — All biological replicates combined. Each dot represents the number of contacts identified in each 1MB segment. Red lines in the lower panels are loess smoothers. Upper panels show the spearman rank correlation between datasets. All correlations are statistically significant (P < 0.001). (TIF) [file pgen.1007258.s009.tif]

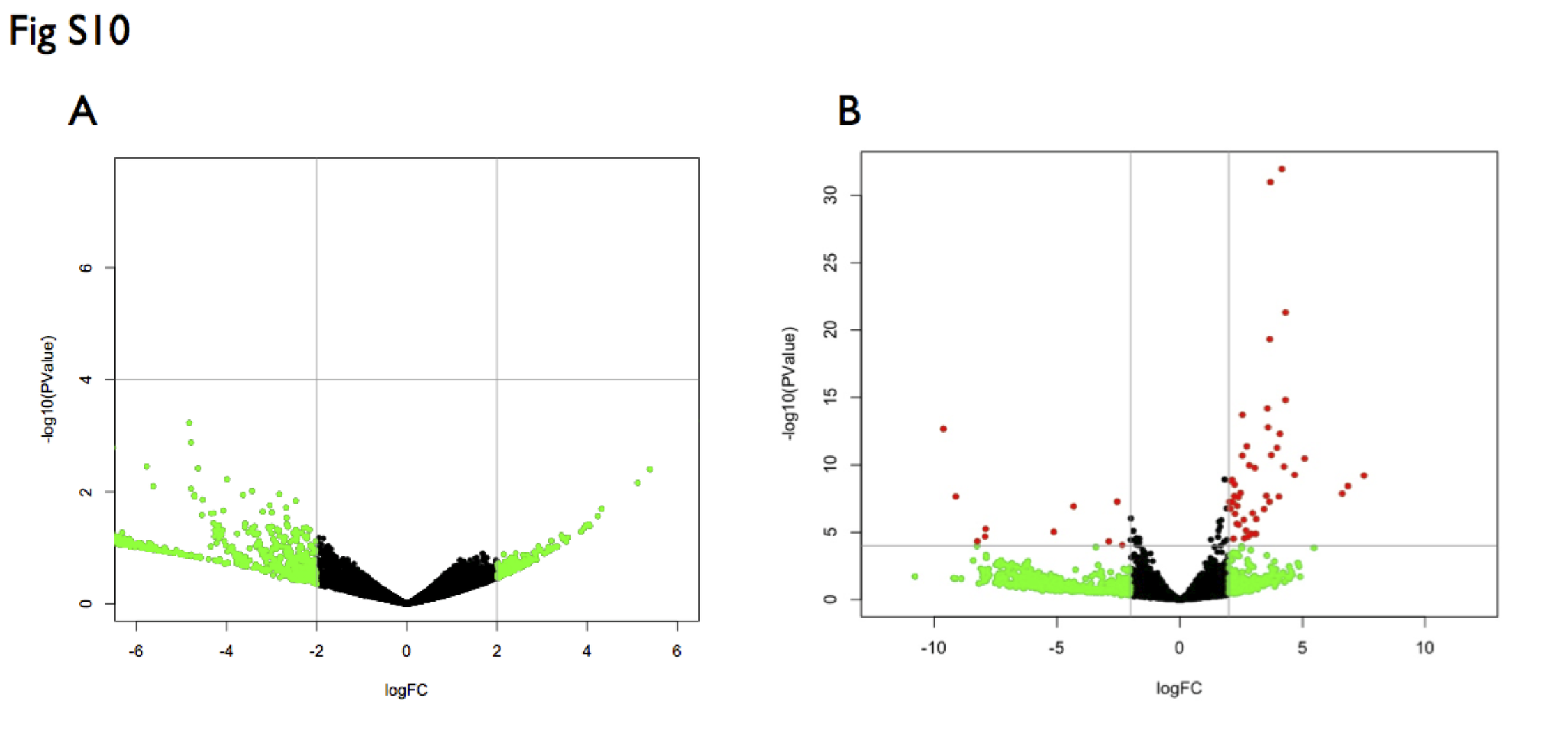

Supplement: S10 Fig — (A) Volcano plot for the contrast between biological replicates of LCL collected with different enzymes (Mbol and DpnII). (B) Volcano plot for the contrast between LCL vs K562 replicates collected with the same enzyme (Mbol). Green dots represent genes with > 2-fold change. Red dots represent segments with difference larger than 2-fold change (vertical line) and P-value < 3 × 10^−4^. (horizontal line). (TIF) [file pgen.1007258.s010.tif]

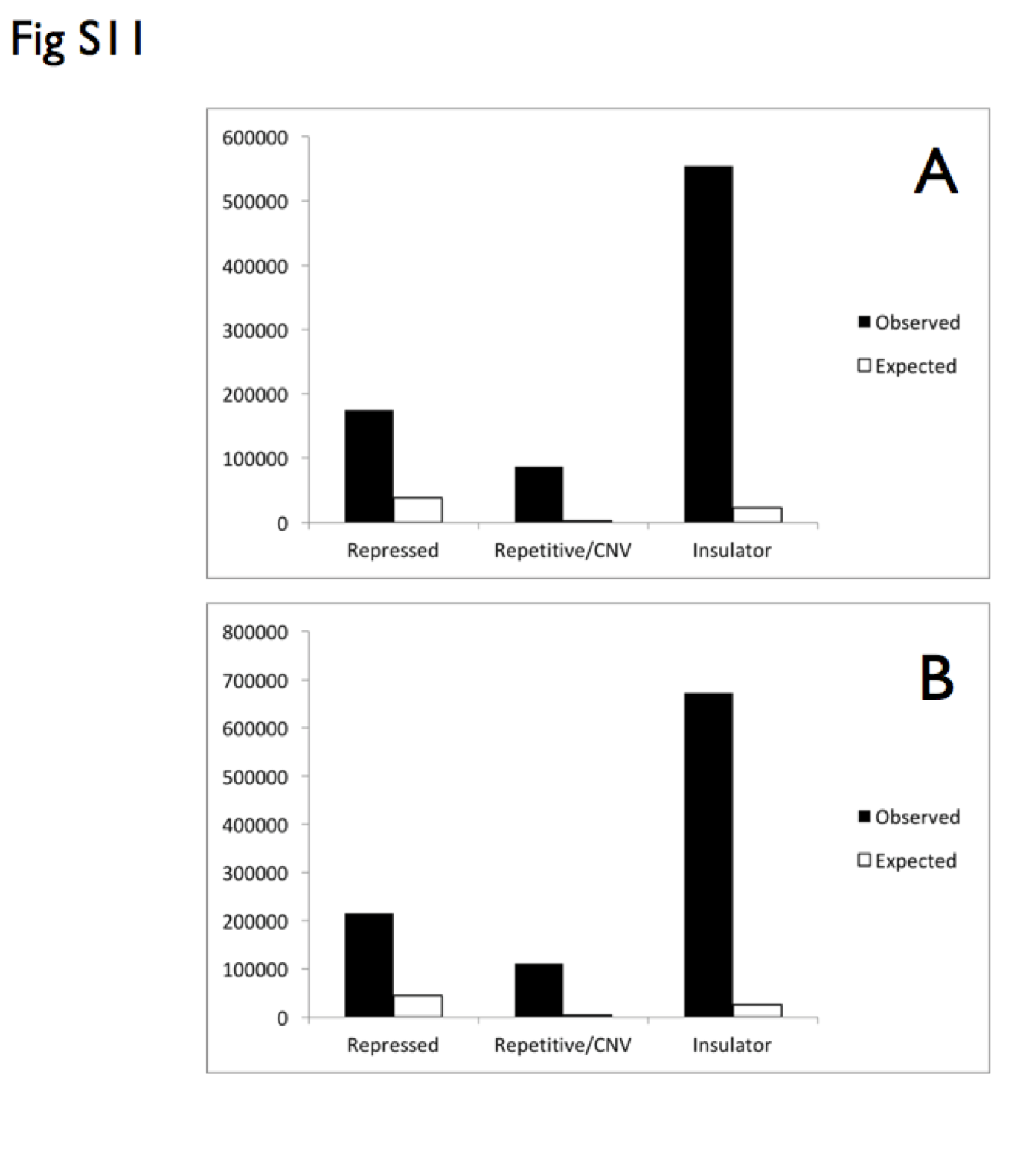

Supplement: S11 Fig — (A and B) Number of observed (black) and expected (white) rDNA contacts with each functional annotation for two sets of biological replicates in LCL. Expected numbers are calculated with the genome-wide per nucleotide contact rate. Shown is data for three selected annotations from 15-label genomic segmentation of hESC using ChromHMM [61]. (TIF) [file pgen.1007258.s011.tif]

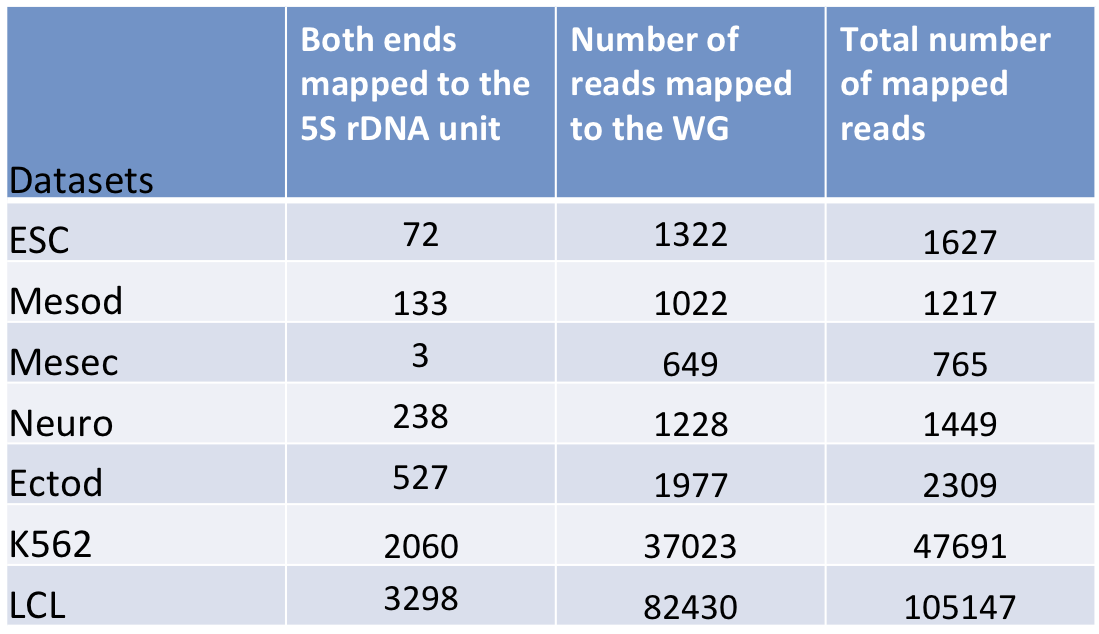

Supplement: S1 Table — Shown are the number of reads for which both ends map to the 5S repeat unit and the number of reads for which one end maps to the 5S rDNA repeat unit and the other maps to the rest of the genome [whole genome (WG)]. Both the 5S rDNA unit and the whole genome were masked for repetitive elements. (TIF) [file pgen.1007258.s012.tif]

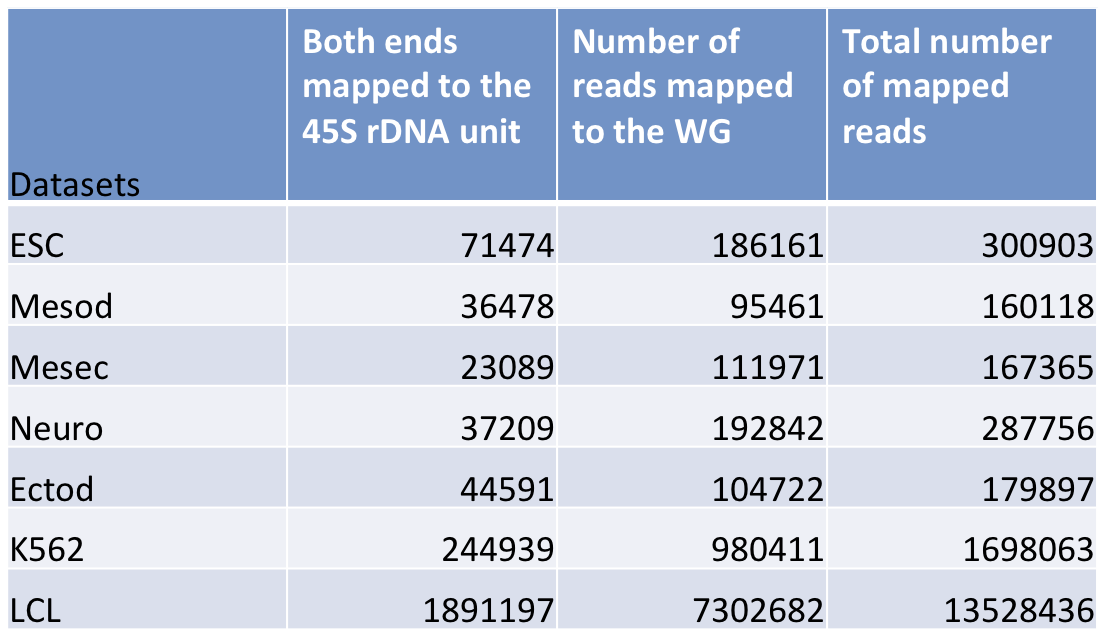

Supplement: S2 Table — Shown are the number of reads for which both ends map to the 45S rDNA repeat unit and the number of reads for which one end maps to the 45S rDNA repeat unit and the other maps to the rest of the genome [whole genome (WG)]. Both the 45S rDNA unit and the whole genome were masked for repetitive elements. (TIF) [file pgen.1007258.s013.tif]

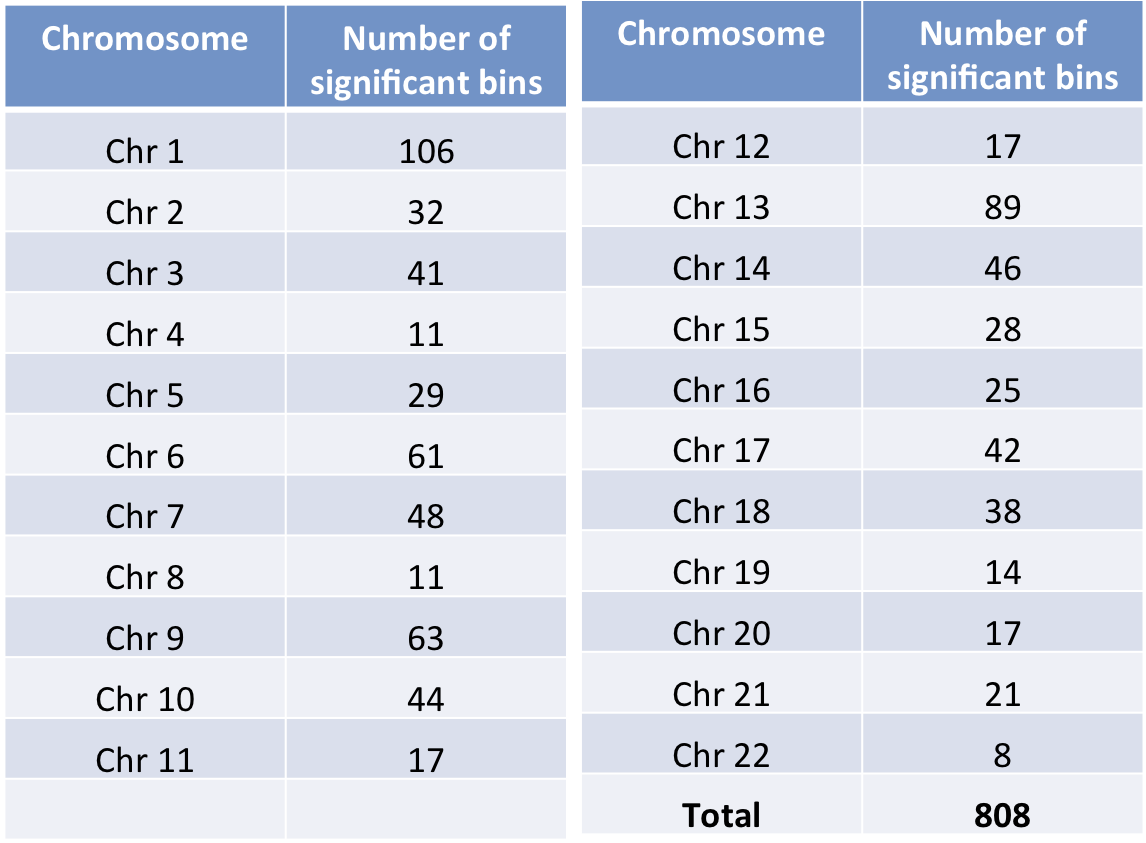

Supplement: S3 Table — Statistically significant segments ascertained with the EdgeR package. The number of segments with significant differences is shown for each chromosome. (TIF) [file pgen.1007258.s014.tif]

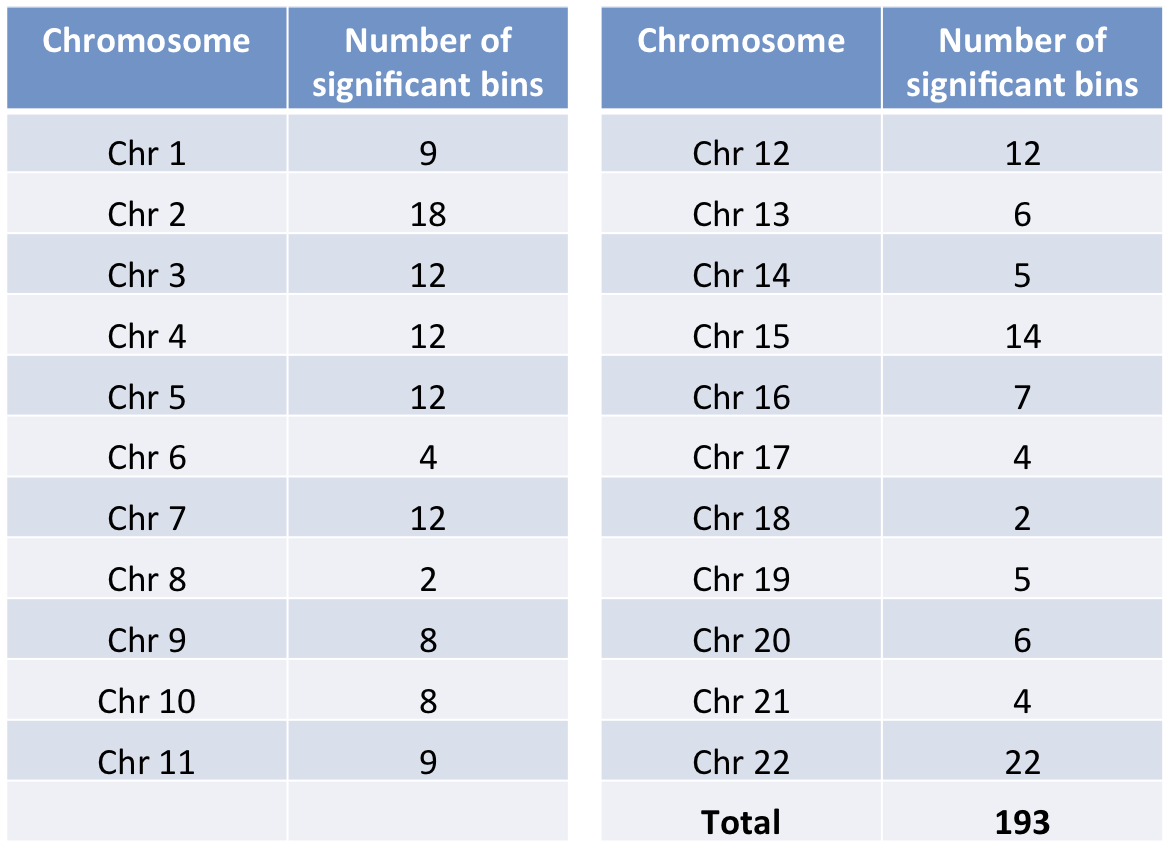

Supplement: S4 Table — Statistically significant segments ascertained with the EdgeR package. The number of segments with significant differences is shown for each chromosome. (TIF) [file pgen.1007258.s015.tif]

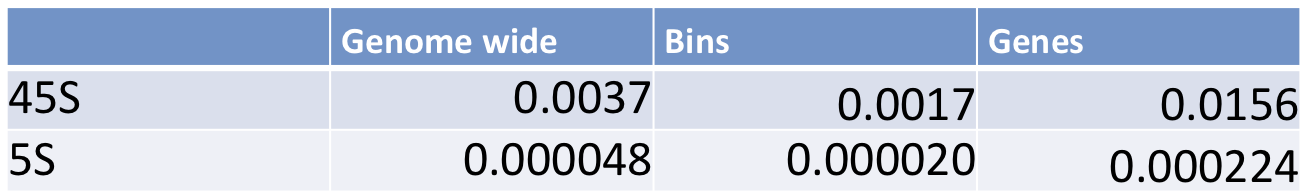

Supplement: S5 Table — CPN obtained from (i) all rDNA-genome contacts (without controlling for pseudogenes), (ii) rDNA-genome contacts retrieved from 1MB bins without rDNA pseudogenes, and (iii) rDNA-gene contacts retrieved from genes without rDNA pseudogenes in them. Genome-wide contacts were divided by the genome size or the total size of the bins without pseudogenes. rDNA-gene contacts were divided by the total length of the genome with genic sequences (protein-coding genes only). Shown are estimates for both 5S and 45S rDNA arrays. (TIF) [file pgen.1007258.s016.tif]

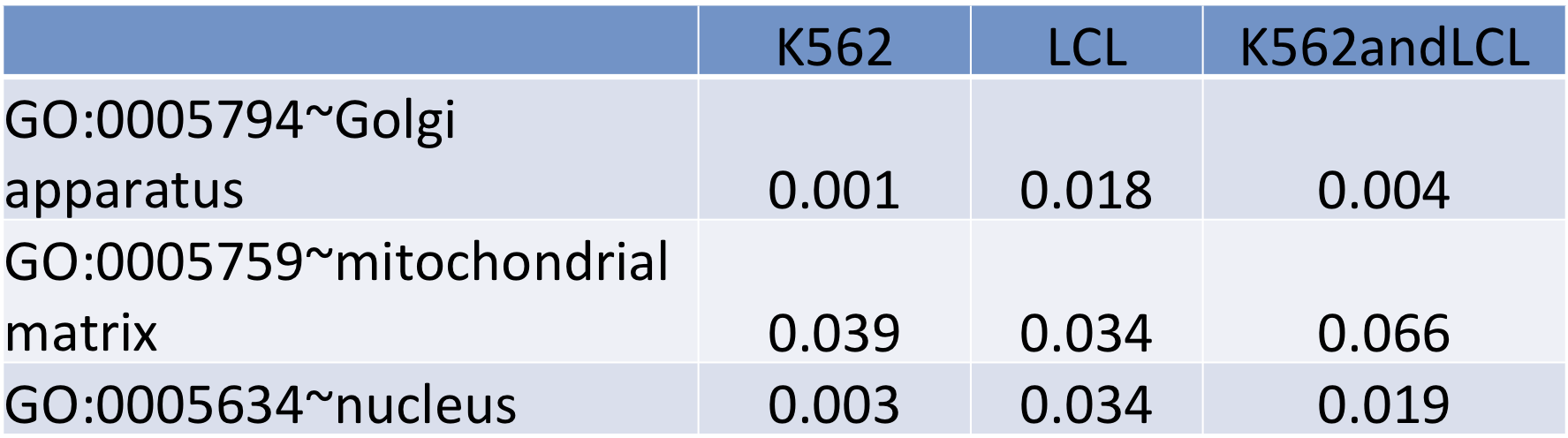

Supplement: S6 Table — Selected categories are shown. (TIF) [file pgen.1007258.s017.tif]

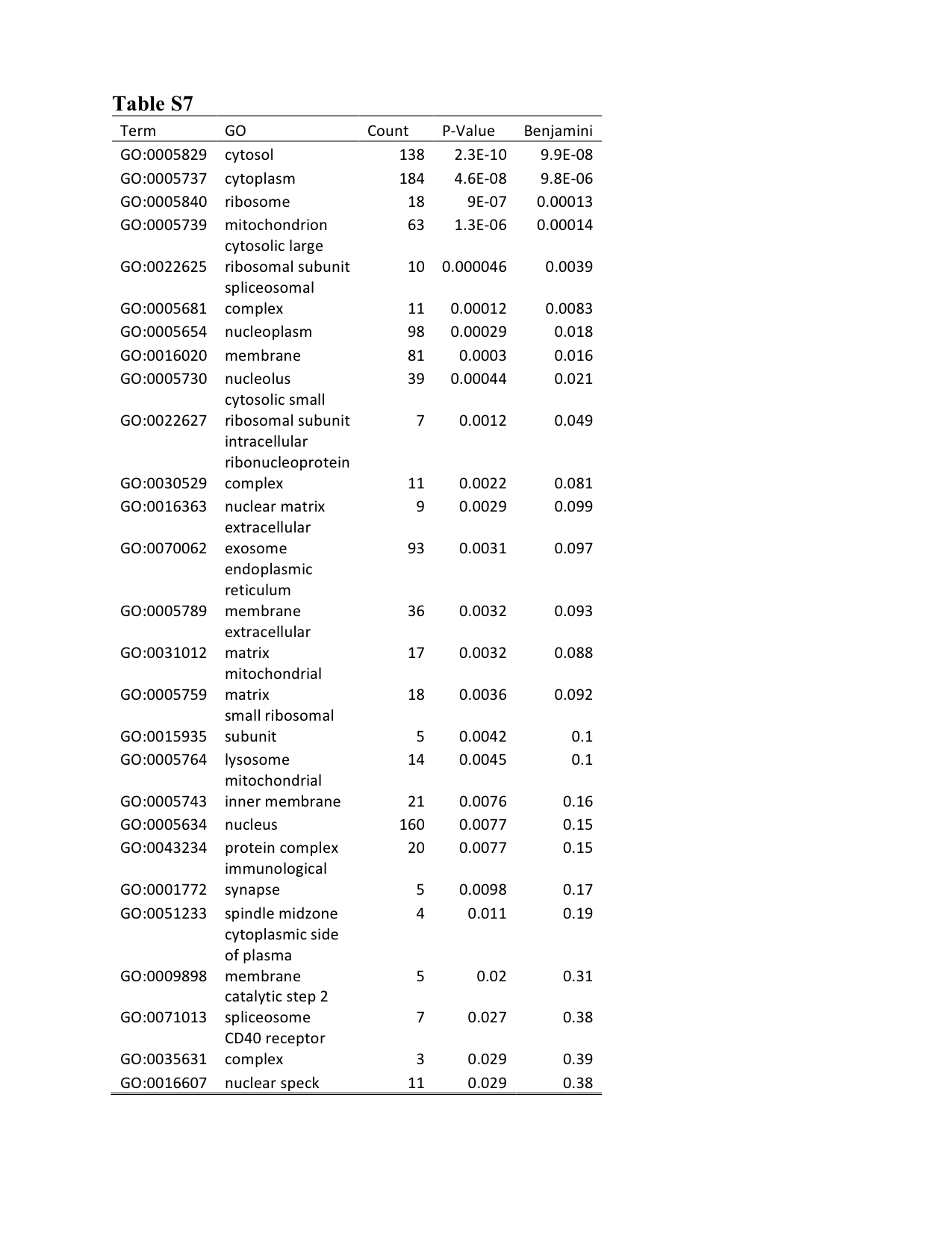

Supplement: S7 Table — Raw and adjusted P-values are listed. Adjusted P-values were obtained after Benjamini-Hochberg correction. All categories with raw P-value < 0.05 are shown. (TIF) [file pgen.1007258.s018.tif]

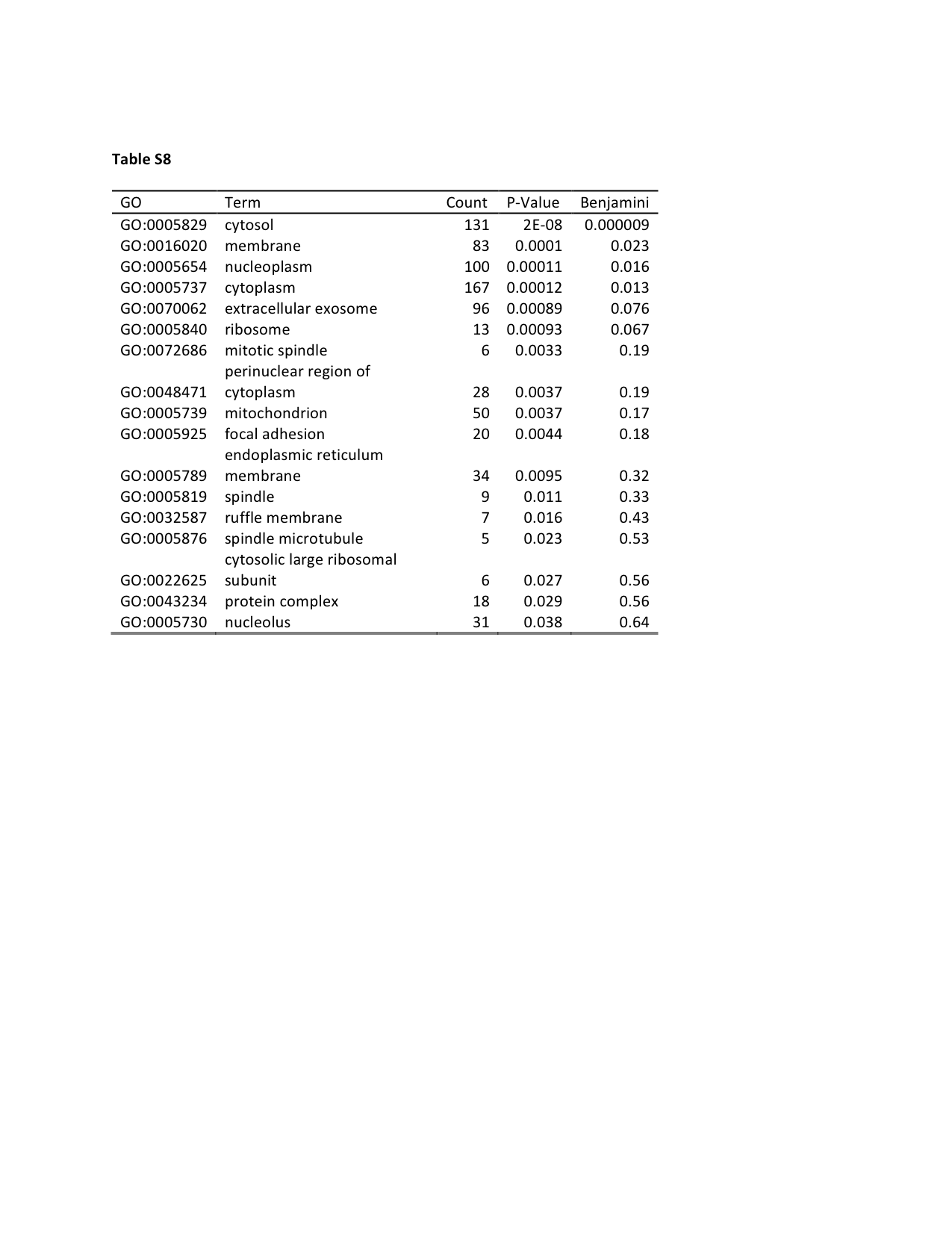

Supplement: S8 Table — Raw and adjusted P-values are listed. Adjusted P-values were obtained after Benjamini-Hochberg correction. All categories with raw P-value < 0.05 are shown. (TIF) [file pgen.1007258.s019.tif]

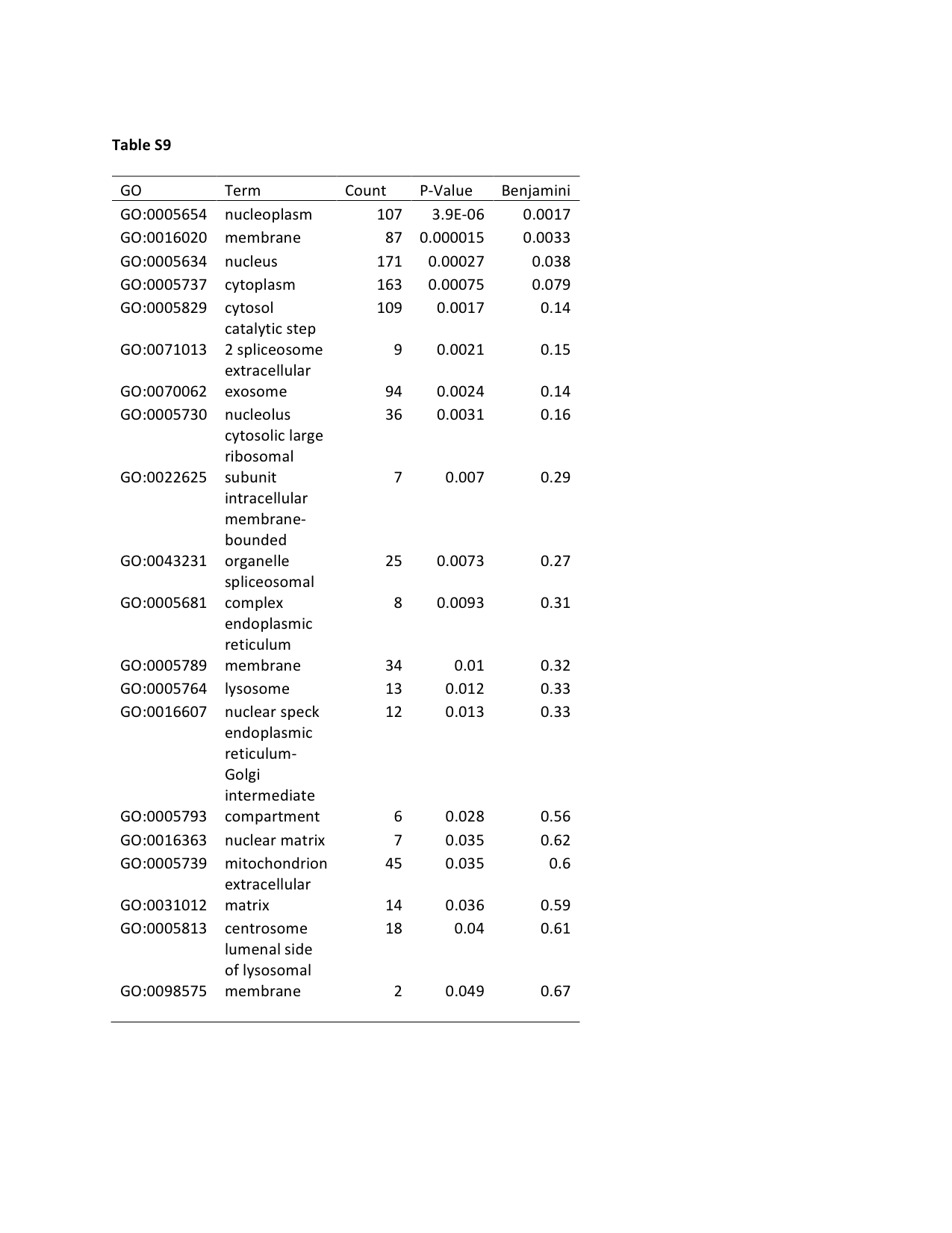

Supplement: S9 Table — Raw and adjusted P-values are listed. Adjusted P-values were obtained after Benjamini-Hochberg correction. All categories with raw P-value < 0.05 are shown. (TIF) [file pgen.1007258.s020.tif]
